# Systematic review and meta-analysis reveal positive therapeutic effects of music in brain damage rehabilitation

**DOI:** 10.3389/fnint.2026.1720473

**Published:** 2026-02-20

**Authors:** Laura Navarro, Nour El Zahraa Mallah, Jacobo Pardo-Seco, Alberto Gómez-Carballa, Sara Pischedda, Wiktor Nowak, Emma Segura, Antoni Rodríguez-Fornells, Federico Martinón-Torres, Antonio Salas

**Affiliations:** 1 Unidade de Xenética, Instituto de Ciencias Forenses, Facultade de Medicina, Universidade de Santiago de Compostela, and Genética de Poblaciones en Biomedicina (GenPoB)Research Group, Instituto de Investigación Sanitaria (IDIS) Hospital Clínico Universitario de Santiago (SERGAS), Galicia, Spain; 2 Genetics, Vaccines and Infections Research Group (GenViP), Instituto de Investigación Sanitaria de Santiago Universidade de Santiago de Compostela, Santiago de Compostela, Galicia, Spain; 3 Centro de Investigación Biomédica en Red de Enfermedades Respiratorias (CIBER-ES), Madrid, Spain; 4 Cognition and Brain Plasticity Unit, Bellvitge Biomedical Research Institute L’Hospitalet de Llobregat, Barcelona, Spain; 5 Institució Catalana de Recerca i Estudis Avançats (ICREA) Barcelona, Spain; 6 Translational Pediatrics and Infectious Diseases, Department of Pediatrics Hospital Clínico Universitario de Santiago de Compostela, Santiago de Compostela, Galicia, Spain

**Keywords:** acquired brain injury, brain damage, meta-analysis, music-based interventions, non-traumatic brain injury, stroke, traumatic brain injury

## Abstract

Brain damage (BD) caused by stroke, traumatic brain injury (TBI), or neurodegenerative conditions often results in persistent cognitive, motor, and emotional impairments. Music-based interventions (MI) have been explored as adjunctive rehabilitation strategies; however, the evidence remains fragmented. This systematic review and meta-analysis synthesize available research on the effects of MI on functional recovery following BD, due to acquired brain injury (ABI), including both TBI and non-TBI. From a total of 868 publications screened in PubMed, Embase, Scopus, Cochrane Library, Web of Science, and ClinicalTrials.gov, 90 were included, of which 41 met the criteria for quantitative evaluation and meta-analysis, to assess the state-of-the-art of research on music and BD in the fields of neuropsychology and cognitive sciences. The reviewed studies span a range of methodologies, including randomized controlled trials and qualitative research, and incorporate diverse MI strategies, such as active music-making, structured listening, and improvisational techniques. The findings indicate that music supports recovery across motor, cognitive, and, albeit to a lesser extent, communicative and psychosocial domains. The findings suggest beneficial effects of MI, particularly in gait function (*z* = 3.46, *P* < 0.01), upper extremity function (*z* = 6.11, *P* < 0.01; UEF), communication (*z* = 3.21, *P* < 0.01), cognitive rehabilitation (*z* = 3.29, *P* < 0.01), and emotional, behavioral, and social outcomes (*z* = 2.35, *P* = 0.02); notably, these effects were often supported by consistent statistical significance across multiple subgroup analyses (e.g., gait, UEF). This study highlights the therapeutic potential of music in neurorehabilitation and supports its integration into multidisciplinary treatment programs. Despite these promising findings, methodological heterogeneity, small sample sizes, and short intervention durations limit the generalizability of results. The evidence suggests that music may modulate key neurobiological pathways in BD, supporting its integration into evidence-based neurorehabilitation programs.

## 1 Introduction

Music is an integral part of daily life across all ages and cultures, serving as one of the most universal forms of expression and communication (Mehr et al., 2019). While cultural and educational perspectives highlight the significance of musical stimuli for human development and wellbeing (Welch et al., 2020), scientific exploration of music’s biological effects remains relatively limited. Music is an extremely complex auditory stimulus, with dynamic changes over time in various acoustic sound features, including timbre, intensity, frequency, and tempo, representing a complex version of the acoustic environment. Over the past two decades, cognitive sciences and neurosciences have shown increasing interest in music as a highly complex and versatile stimulus, offering valuable insights into brain functions (Koelsch, 2015; Salimpoor et al., 2015). Relevant studies have demonstrated the plasticity of the human auditory cortex in response to musical training (Pantev and Herholz, 2011). Musical processing engages a widespread network of brain structures beyond the auditory cortex, encompassing areas involved in motor, cognitive, language, emotional and reward processing, including the cerebellum, planum temporale, parietal lobe, insula, limbic circuit, ventral striatum (nucleus accumbens), ventral tegmental area, premotor cortex, anterior superior-temporal gyrus, and frontal lobe, among others (Lin et al., 2011). From a genetic perspective, research has identified specific genomic regions and specific genes associated with musical traits (Liu et al., 2016), examined the heritability of various musical characteristics (de Manzano and Ullén, 2018), and explored the effects of music on gene expression (Gómez-Carballa et al., 2023) and on the microbiome (Cavenaghi et al., 2025), with the latter two studies conducted within the framework of the sensogenomics project (Navarro et al., 2021; Salas et al., 2025).

Stroke has been identified as the leading contributor to neurological diseases in a recent report, with its incidence increasing by 86.1% from 1990 to 2021 (GBD 2021 Nervous System Disorders Collaborators, 2024). Brain damage (BD), whether resulting from stroke, traumatic brain injury (TBI), or other neurological issues, is a leading cause of long-term disability worldwide. Survivors often experience ongoing challenges in motor skills, cognitive abilities, emotions, and social interactions, which can really affect their independence and quality of life (QoL). Thus, BD, as both a health and social issue, demands innovative approaches to explore the potential of non-pharmacological interventions in improving patients’ QoL. Yet, despite advances in neurorehabilitation, many individuals achieve only partial recovery, which underscores the need for adjunctive therapies that can enhance functional outcomes. In this context, music-based interventions (MI) have gained increasing attention in neurorehabilitation due to the multimodal stimulation provided by music, which is key to support neural recovery in neurodegenerative conditions (Navarro et al., 2023) and in BD following stroke or other acquired brain injuries (ABI).

Musical activities, such as listening, instrumental playing, or singing, simultaneously engage auditory, sensorimotor, cognitive, language, visual, and emotional systems. Functional recovery relies on learning-driven neural plasticity, which is supported by interactions among reward, attention, and memory networks (Carey, 2012; Dayan and Cohen, 2011). Music engagement requires higher-order cognitive processes, including attention and working memory, to process timing, melody, and lyrics. These processes are mediated by prefrontal, cingulate, and parietal cortices, which interact with auditory and motor systems through dopaminergic pathways. Such interactions enhance sensory processing, facilitate memory consolidation, and support compensatory motor, cognitive, and language functions (Jerde et al., 2011; Posner et al., 2014; Schulze et al., 2011; Talsma et al., 2010). Auditory and motor systems are also linked to limbic regions through dopaminergic pathways, eliciting affective and reward responses that regulate mood and reinforce motivation to engage in music (Ferreri et al., 2019; Salimpoor et al., 2009; Salimpoor et al., 2011; Zatorre and Salimpoor, 2013). Through this dopaminergic modulation, music engagement can promote lasting functional changes in auditory, motor, and associative cortices (Hosp et al., 2011).

In neurorehabilitation, MI aim to restore impaired functions by recruiting preserved brain regions that facilitates neural recovery (Sihvonen et al., 2017a; Wan et al., 2010). Motor recovery is supported by auditory-motor coupling, which aids movement planning and execution through intact auditory pathways (Sihvonen et al., 2017a). Instrument playing engages multiple motor regions that are supported by continuous auditory feedback, enabling precise motor control while stimulating cognitive processes (Grau-Sánchez et al., 2020). For speech and language recovery, singing- and rhythm-based interventions engage shared neural networks for timing and prosody, enhancing articulation, fluency, and word retrieval by recruiting unaffected brain regions (Norton et al., 2009; Patel, 2011). MI can be classified as receptive (e.g., music listening) or active (e.g., music making) (Sihvonen et al., 2017a). While receptive approaches haven been shown to primarily improve cognition and mood (Särkämö and Soto, 2012; Särkämö et al., 2008), active MI techniques—such as rhythmic auditory stimulation, melodic intonation therapy, and music-supported therapy—have demonstrated broader benefits, including enhanced motor plasticity, language recovery, cognitive performance, and emotional wellbeing (Bernardi et al., 2017; Fujioka et al., 2018; Marchina et al., 2023; Ripollés et al., 2016). Furthermore, previous reviews, mostly systematic or narrative (qualitative), with only a few including meta-analyses on specific outcomes, have focused on the rehabilitative effects of music, particularly examining specific outcomes such as the effectiveness of music therapy in TBI (Mishra et al., 2021), the effects of choral singing on BD (Monroe et al., 2020), the benefits of active MI in upper-limb rehabilitation after stroke (Grau-Sánchez et al., 2020), or the impact of previous musical training following BD (Omigie and Samson, 2014). A key systematic review (Magee et al., 2017) comprehensively analyzed the effects of MI on BD, while other reviews have focused on specific outcomes, such as gait improvement (Ghai, 2023) or language recovery (Yang et al., 2019). This suggests that music can do more than just lift our spirits; it might actually help reorganize damaged neural pathways, offering both emotional support and motivation along the way.

With variability in intervention types, duration, timing, and outcome measures, findings of previous work have been mixed. Consequently, our study presents the first comprehensive and integrative systematic review and meta-analysis on the multifaceted impact of MI in BD rehabilitation. By bridging motor, cognitive, and wellbeing domains, we synthesize a wide range of research through both qualitative analysis and quantitative meta-analytic methods. This dual approach not only deepens interpretive insight but also offers robust statistical evidence, shedding new light on the complex and often underestimated interplay between music and neurological recovery.

## 2 Materials and methods

### 2.1 Methodological aspects of the systematic review

A systematic review was conducted using the PubMed database, with the initial search covering the period from 1 January 2000 to 24 March 2023. To ensure comprehensive coverage of literature relevant to BD, we selected search terms such as “brain injur*” (which considers both terms, injury and injuries), “brain deformation,” and “cerebral damage,” based on their frequency and relevance in the literature. The term “music” was intentionally kept broad to avoid limiting the scope of the study. We applied the [tiab] field tag (title and abstract) to restrict results to articles containing these specific terms, thereby filtering out irrelevant publications. The final search query was as follows: (“brain injur*”[tiab] OR “brain damage*”[tiab] OR “brain deformation*” [tiab] OR “cerebral injur*” [tiab] OR “cerebral damage*” [tiab]) AND (music*[tiab]). This initial search yielded 247 records. These were refined by removing duplicates, systematic reviews, and meta-analyses using PubMed’s built-in filters. Additional filters were applied to include only English-language studies focused on human subjects. From the remaining articles, 13 were excluded manually for being reviews or systematic reviews.

Furthermore, a detailed screening was done based on predefined inclusion and exclusion criteria, where studies were excluded if they: (a) involved mixed therapeutic approaches incorporating music alongside other interventions, as these could confound the isolated effects of MI; (b) were not directly related to the impact of music on ABI; (c) the patients group was not homogeneous; (d) not focusing on a rehabilitation program; or (e) is not based on an experimental design. We also excluded one article focused on congenital brain disorders, as this review specifically targets ABI. These criteria were established to guarantee conceptual clarity and to enhance the validity of the synthesis, following best-practice recommendations for systematic reviews as outlined by Shea et al. (2017).

**FIGURE 1:**
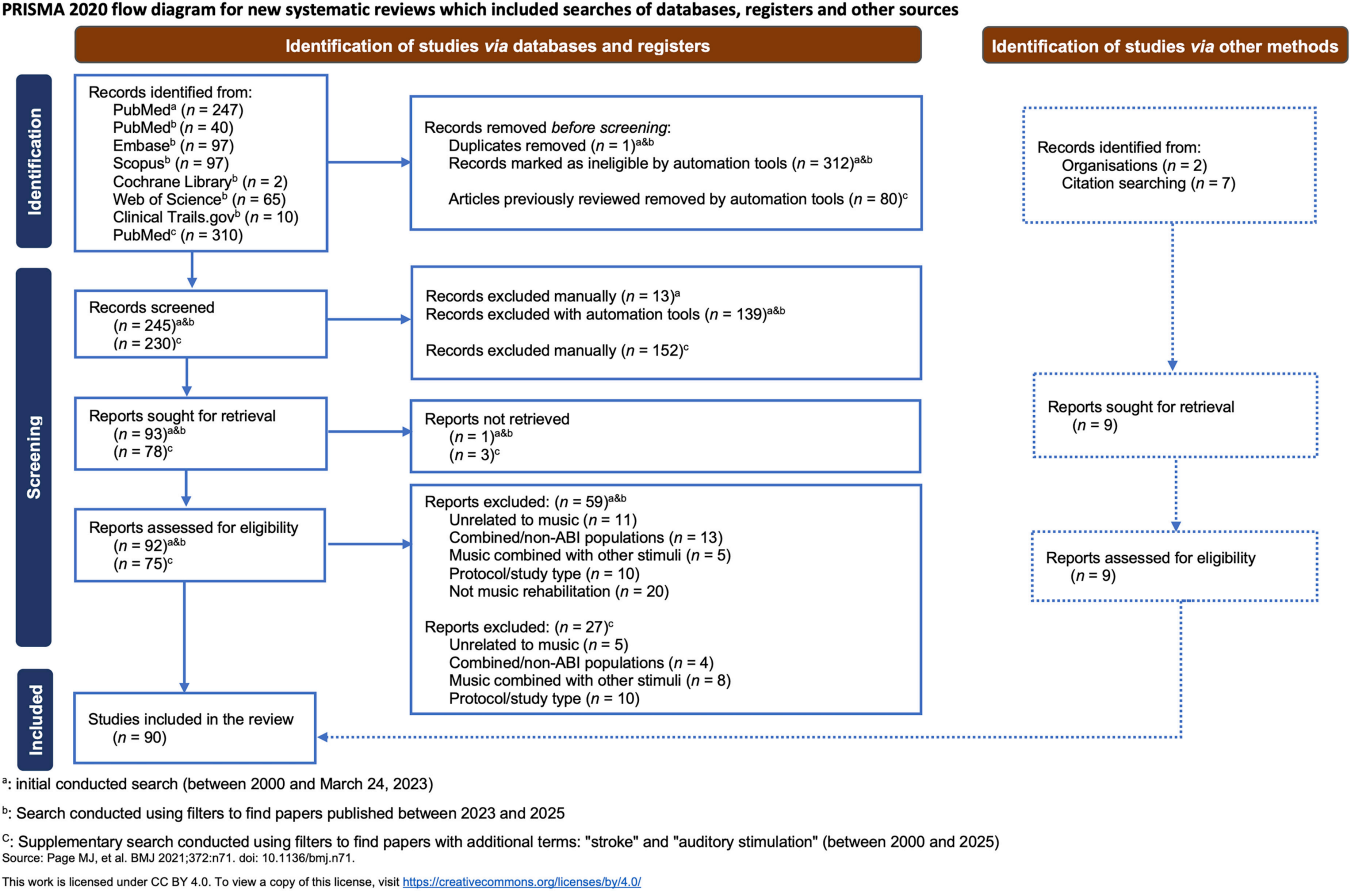
PRISMA flow diagram of study selection. This diagram outlines the systematic identification, screening, eligibility assessment, and final inclusion of studies in accordance with PRISMA guidelines. The process includes records identified *via* PubMed and citation searching, detailing the number of studies excluded at each stage and the reasons for exclusion. The flowchart follows the updated PRISMA framework proposed by Page et al. (2021).

To ensure the relevance and completeness of the review, a second search was conducted on June 11, 2025. This search was extended to multiple databases, including PubMed, Embase, Scopus, the Cochrane Library Core Collection, Web of Science, and ClinicalTrials.gov. The same search terms and strategy were used to maintain consistency, with filters applied to restrict results to studies published between 2023 and 2025. This yielded a total of 311 results across all platforms. Duplicates were removed manually or using the automation tool Rayyan (ryyan.ai). The remaining articles were screened manually in accordance with the inclusion/exclusion criteria described above (Figure 1).

Since some studies did not include the term “brain damage/injury”, even though the participants were stroke patients, an additional search was conducted that included the missing terms “stroke” and “auditory stimulation” to ensure the completeness and robustness of our review. This third query resulted in 310 additional articles that were screened. Using Rayyan, 80 duplicates were automatically removed. Out of the resulting 230 articles, 152 were manually excluded, yielding 78 unique papers. After the manual screening, a total of 20 additional papers were included (Figure 1). Moreover, 9 relevant articles were incorporated following an expert consultation or through reference mining of previous reviews and other key sources. Accordingly, a total of 90 articles were included in this review; Supplementary Table 1.

This review was conducted and reported in agreement with the Preferred Reporting Items for Systematic Reviews and Meta-Analyses (PRISMA) guidelines^1^ (see Figure 1). Furthermore, we utilized the JBI Critical Appraisal Tool to evaluate the risk of bias of the included studies, based on their type (Barker et al., 2023; Barker et al., 2024; Supplementary Table 2).

### 2.2 Meta-analysis

Complementing the qualitative synthesis, this review incorporates a meta-analysis of 41 studies that provided complete data suitable for statistical evaluation. The analysis targets key outcomes frequently assessed in BD research, including general improvement, motor recovery, communication, cognitive rehabilitation, QoL, and emotional wellbeing. A standardized approach was employed to synthesize findings across these diverse domains, ensuring methodological rigor. Data extraction was conducted independently by two reviewers. Studies were excluded if they lacked a control group, focused exclusively on acute physiological outcomes, or did not isolate the effects of music from other concurrent interventions.

Extracted data included sample sizes, means, and standard deviations (SDs) for both experimental and control groups, the type of outcome measure used, intervention duration, and study design characteristics. For each outcome, we estimated the pooled mean difference (MD) and corresponding 95% confidence intervals (CIs) in change scores between baseline and follow-up, comparing the intervention group to non-interventional controls. This was performed using a random-effects meta-analysis, based on DerSimonian and Laird (1986). Although a random-effects model yields wider CIs (accounting for uncertainty due to between-study variability), it is the recommended approach when heterogeneity is moderate to high (as confirmed in several analyses in this study, with *I*^2^ > 50% in some domains) or when studies differ significantly in design, participant characteristics, and intervention methods. Given the considerable heterogeneity in MI procedures for brain-damaged populations, the random-effects model provides a more appropriate and reliable estimation of aggregate effects. Therefore, its use enhances the validity and generalizability of the findings across diverse clinical contexts.

Between-study heterogeneity was evaluated using the I^2^ statistic, tau-squared (τ^2^), and Cochran’s Q test. Subgroup analyses were conducted to explore potential sources of heterogeneity, and subgroup differences were tested using chi-squared (χ^2^) statistics. Forest plots were generated to display both individual study outcomes and pooled effect estimates.

Regarding subgroup and sensitivity analyses, we carefully evaluated the feasibility of performing additional analyses stratified by intervention type, etiology, treatment duration, and musical characteristics. However, the substantial heterogeneity in outcome measures and neuropsychological assessment tools across the included studies markedly limited the possibility of meaningfully grouping studies into homogeneous subcategories. In many instances, the number of studies available within potential subgroups was insufficient to support statistically robust subgroup or sensitivity analyses.

For each subgroup and the overall analysis, outliers were identified based on the distribution of study-level effect estimates and the lengths of their confidence intervals (CIs). Studies were flagged if their effect estimate or CI length lay outside the range defined by *Q1* − 1.5 × *IQR* or *Q3* + 1.5 × *IQR* (Interquartile Range, IQR). Studies meeting this criterion for either the effect estimate or the CI length, or presenting missing confidence intervals, were excluded from the corresponding sensitivity analyses. These exclusions were outcome-specific and applied only when a minimum number of studies (≥ 2) remained available for comparison.

These procedures were applied solely for sensitivity analyses and not for permanent exclusion from the study. Robustness was assessed by comparing results from the primary meta-analysis, which included all eligible studies, with those obtained after excluding flagged studies, with consistency of effect estimates used as the criterion for robustness (Table 1).

**TABLE 1:** Comparison of meta-analytic results obtained using the complete set of included studies and after exclusion of studies identified as outliers.

Study	Complete set of studies							Excluding outlier studies						
	*n*	Effect	95% CI	*Z*	*P*	τ^2^	*I*^2^ (%)	*n*	Effect	95% CI	*Z*	*P*	τ^2^	*I* ^2^
[1]	26	0.89	[0.36, 1.42]	3.29	**< 0.01**	0.3	40	21	0.86	[0.34, 1.42]	3.22	**< 0.01**	0.29	45
[2]	6	0.91	[0.3, 1.52]	2.93	**< 0.01**	0	0	4	1	[0.24, 1.52]	2.59	**0.01**	0	0
[3]	4	2.58	[0.89, 4.28]	2.99	**< 0.01**	0	0	3	2.74	[0.99, 4.28]	3.06	**< 0.01**	0	0
[4]	27	3.24	[1.26, 5.22]	3.21	**< 0.01**	14.81	73	24	3.52	[2, 5.22]	4.55	**< 0.01**	5.36	73
[5]	17	0.19	[0.03, 0.35]	2.35	**0.02**	0	52	15	0.19	[0.03, 0.35]	2.32	**0.02**	0	52
[6]	5	4.44	[−1.56, 10.43]	1.45	0.15	34.06	73	4	−0.02	[−0.42, 10.43]	−0.08	0.15	0	73
[7]	27	3.24	[1.26, 5.22]	3.21	**< 0.01**	14.81	73	24	3.52	[2, 5.22]	4.55	**< 0.01**	5.36	73
[8]	23	3.76	[1.63, 5.89]	3.46	**< 0.01**	23.87	94	20	2.54	[0.95, 5.89]	3.12	**< 0.01**	11.51	95
[9]	4	8.44	[−0.55, 17.43]	1.84	0.07	73.56	95	3	7.66	[−3.73, 17.43]	1.32	0.19	97.84	97
[10]	5	0.16	[0.11, 0.21]	6.46	**< 0.01**	0	22	3	0.17	[0.12, 0.21]	6.55	**< 0.01**	0	0
[11]	49	3.36	[2.28, 4.44]	6.11	**< 0.01**	4.92	49	43	3.39	[2.3, 4.44]	6.12	**< 0.01**	5	47
[12]	8	5.79	[2.79, 8.79]	3.78	**< 0.01**	0	0	5	5.98	[2.88, 8.79]	3.79	**< 0.01**	0.07	0
[13]	4	3.06	[0.27, 5.85]	2.15	**0.03**	0	0	3	3.31	[0.45, 5.85]	2.27	**0.02**	0	0
[14]	4	1.15	[0.16, 2.14]	2.29	**0.02**	0	0	3	1.15	[0.16, 2.14]	2.29	**0.02**	0	0
[15]	5	0.65	[−3.4, 4.7]	0.31	0.75	4.96	0	3	6.29	[−1.8, 4.7]	1.52	0.13	0	0
[16]	3	1.22	[0.53, 1.91]	3.48	**< 0.01**	0	0	2	1.22	[0.53, 1.91]	3.48	**< 0.01**	0	0

**FIGURE 2:**
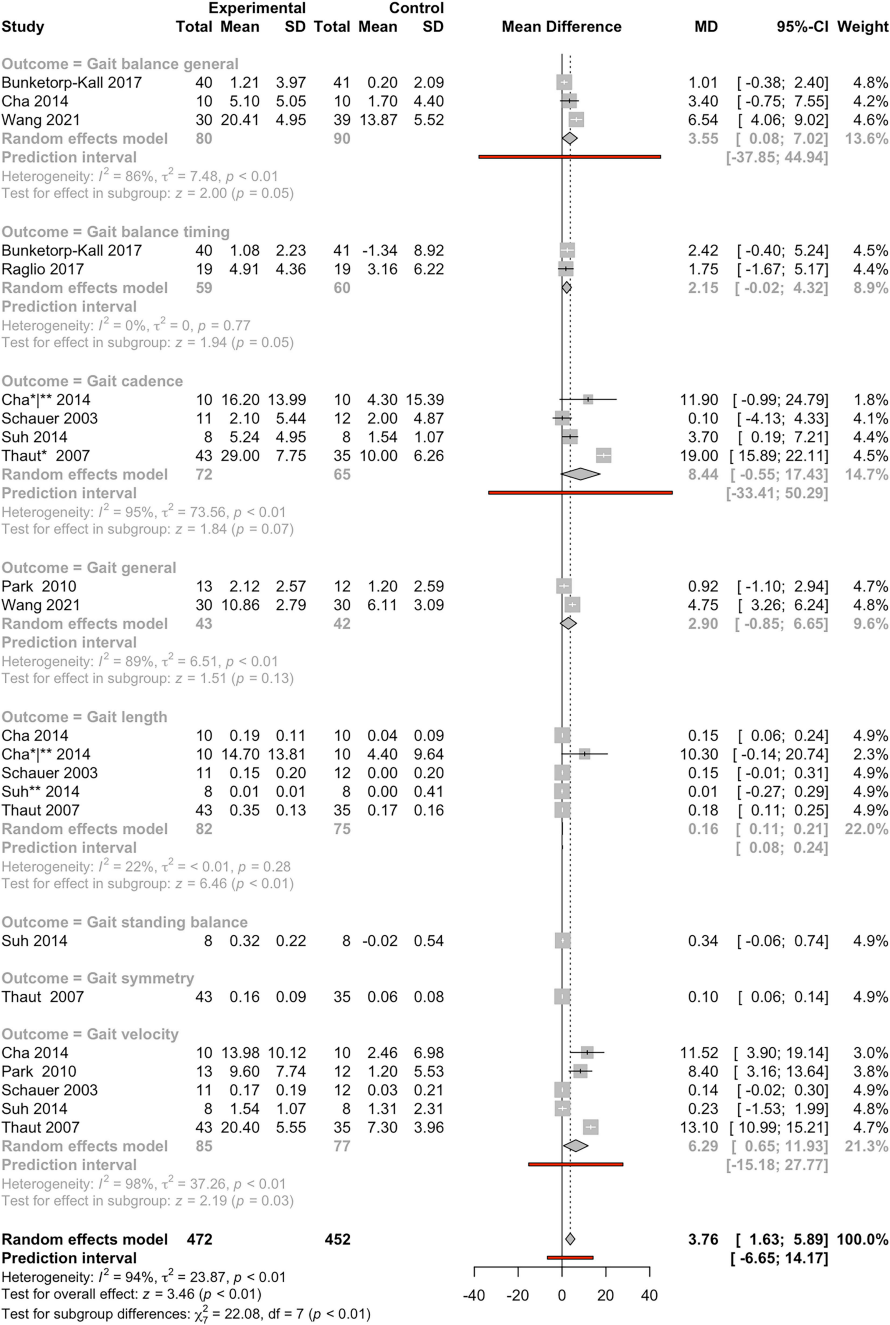
Forest plot of mean differences (MD) between experimental and control groups across studies evaluating the effects of intervention on gait-related outcomes. The plot presents individual study results with corresponding MD, 95% confidence intervals (CIs), and study weights. Summary estimates were calculated using a random-effects model. Heterogeneity statistics (I^2^, τ^2^, and *P*-values) are reported for each outcome, along with prediction intervals and tests for subgroup and overall effects. Outlier studies excluded in the sensitivity analysis are indicated with one asterisk (denoting outliers identified in subgroup-level analyses) or two asterisks (denoting outliers identified in the overall outcome analysis); see Methods for the definition of outliers.

**FIGURE 3:**
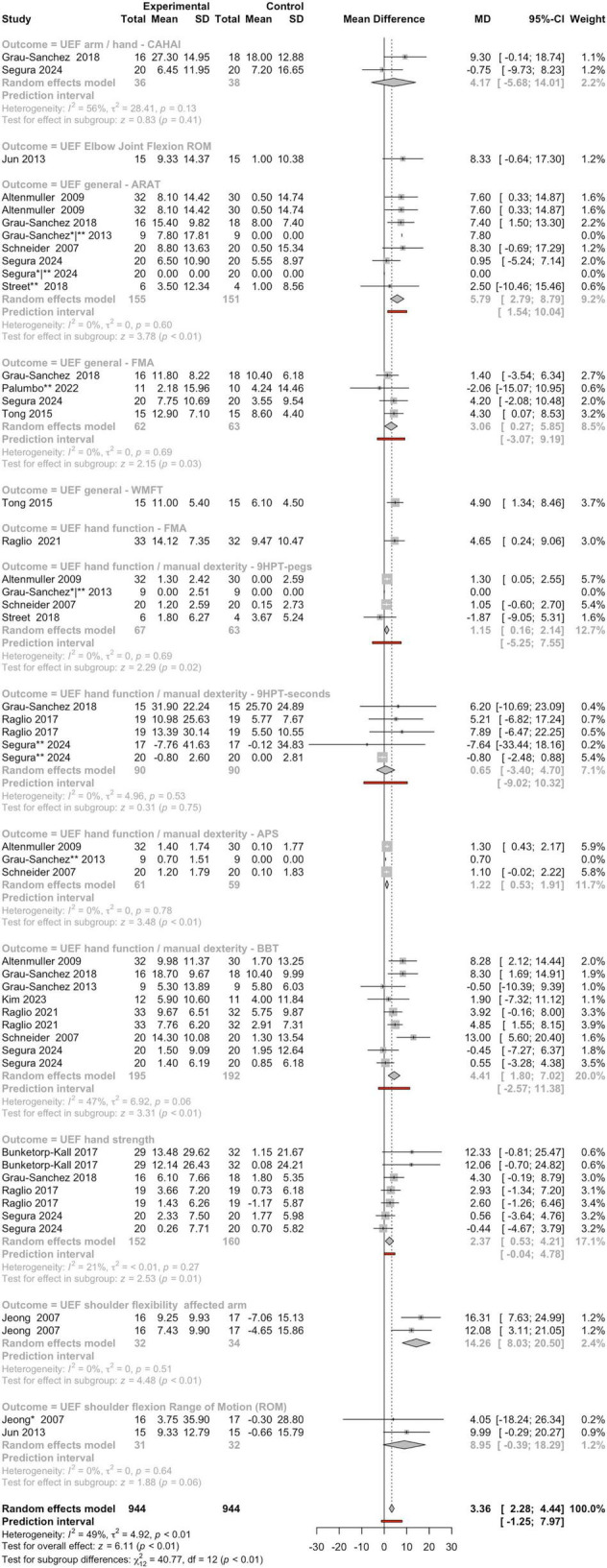
Forest plot of mean differences between experimental and control groups across studies evaluating the effects of intervention on UEF-related outcomes. See legend of Figure 2 for more details.

**FIGURE 4:**
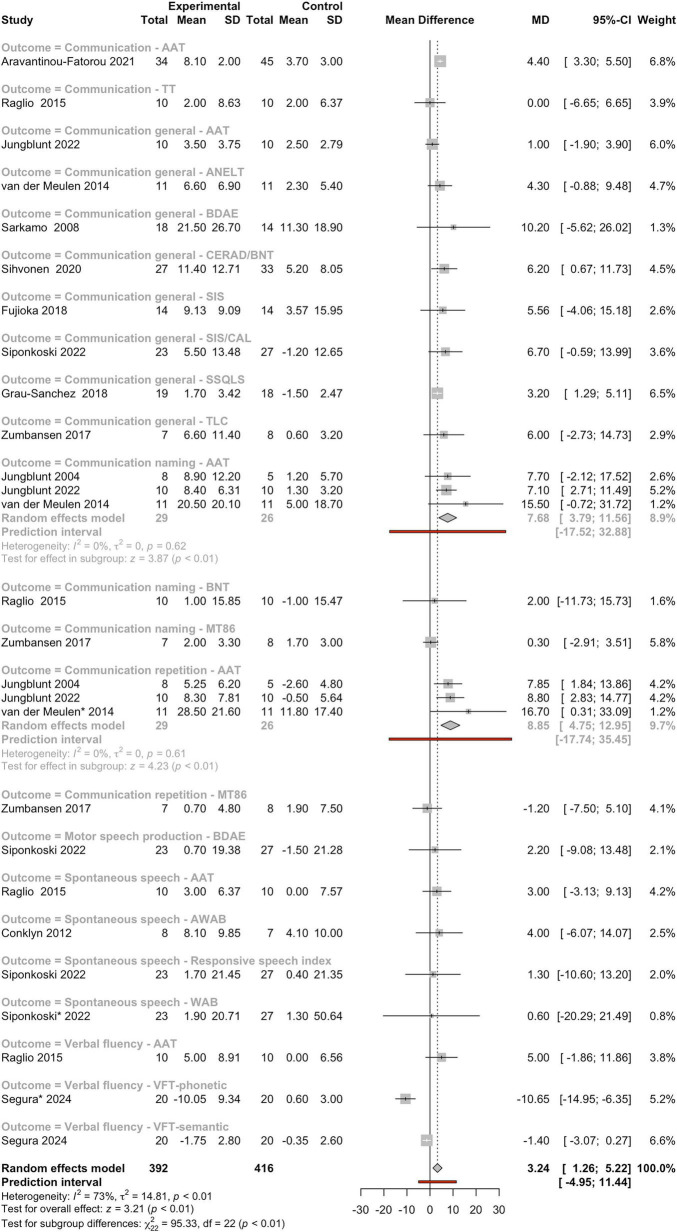
Forest plot of mean differences between experimental and control groups across studies evaluating the effects of intervention on communication-related outcomes. See legend of Figure 2 for more details.

**FIGURE 5:**
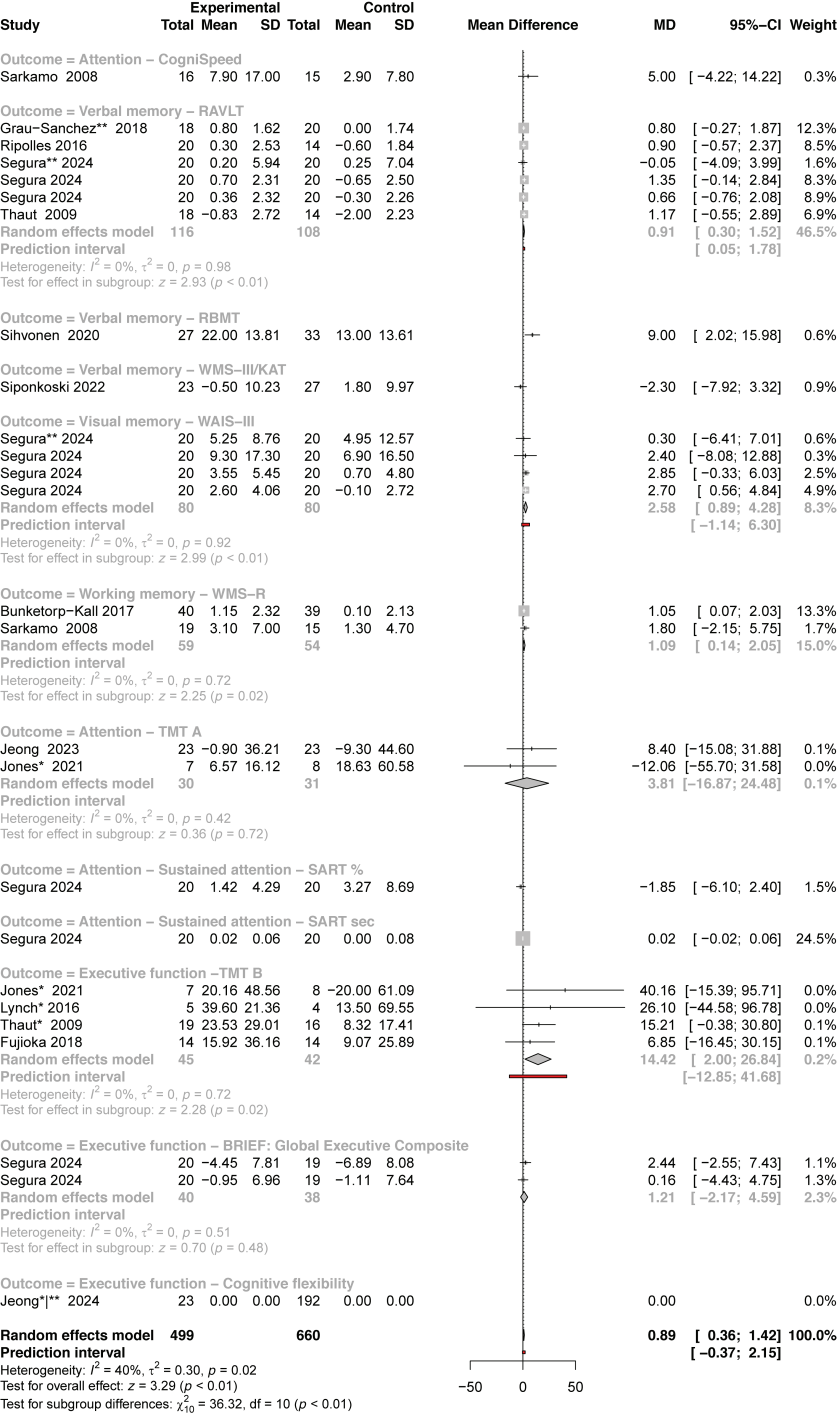
Forest plot of mean differences between experimental and control groups across studies evaluating the effects of intervention on cognitive-related outcomes. See legend of Figure 2 for more details.

**FIGURE 6:**
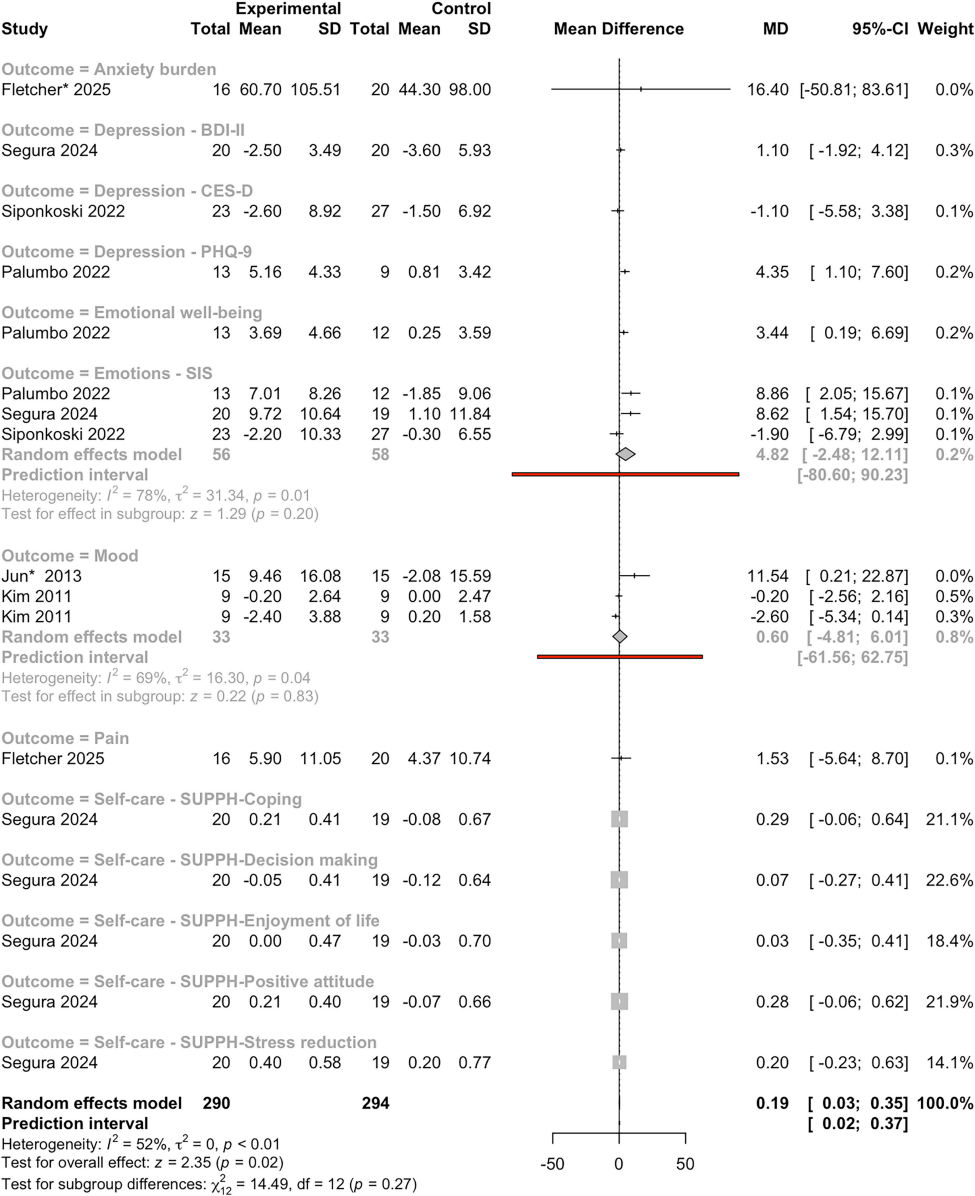
Forest plot of mean differences between experimental and control groups across studies evaluating the effects of intervention on emotional, behavioral and social outcomes. See legend of Figure 2 for more details.

**FIGURE 7:**
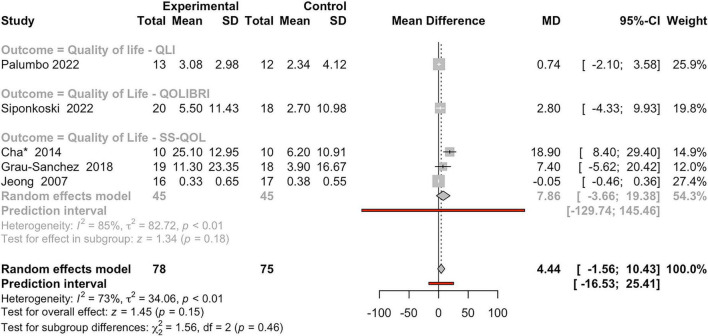
Forest plot of mean differences between experimental and control groups across studies evaluating the effects of intervention on QoL-related outcomes. See legend of Figure 2 for more details.

**FIGURE 8:**
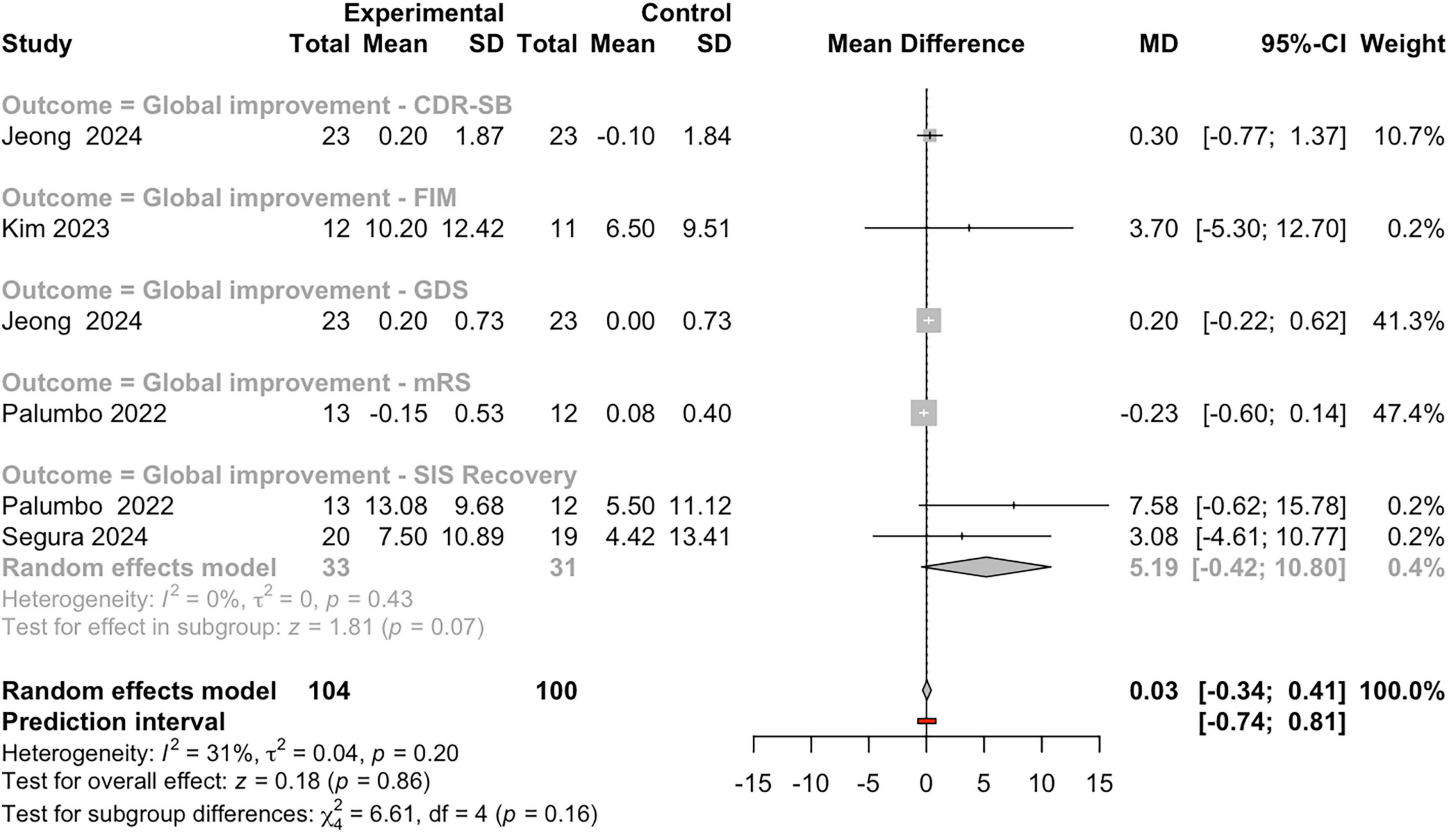
Forest plot of mean differences between experimental and control groups across studies evaluating the effects of intervention on global improvement outcomes. See legend of Figure 2 for more details.

For each main category and eligible subcategory (≥ 2 studies), pooled effect estimates, statistical significance, and heterogeneity measurements are reported for both analytical scenarios. Only subcategories containing outlier studies are indicated. This comparison assesses the robustness of the meta-analytic findings to the influence of outlier studies. Note that all tests statistically significant in the complete set are also statistically significant when excluding outliers (bolded values). Outlier studies are indicated in Figures 2–8. *n* = number of studies included; [1] Cognitive—Overall; [2] Verbal memory—RAVLT; [3] Visual memory—WAIS-III; [4] Communication—Overall; [5] Emotional states—Overall; [6] QQL—Overall; [7] Communication—Overall; [8] Gait—Overall; [9] Gait cadence; [10] Gait length; [11] UEF—Overall; [12] UEF general—ARAT; [13] UEF general—FMA; [14] UEF hand function/manual dexterity—9HPT-pegs; [15] UEF hand function/manual dexterity—9HPT-s; [16] UEF hand function/manual dexterity—APS.

Therefore, in addition to overall estimates, subgroup analyses were also stratified by outcome type [e.g., Fugl-Meyer Assessment (FMA), Action Research Arm Test (ARAT), Box and Block Test (BBT)]. This approach reflects the multidimensional nature of Upper Extremity Function (UEF) assessment and allows the identification of scale-specific variations in intervention effects.

For a small number of studies, measures of dispersion were reported as IQRs or 95% CIs rather than SDs. To ensure consistency of variance metrics across studies and to enable quantitative synthesis, these measures were converted to SDs using established statistical relationships. Specifically, IQRs were converted to SDs assuming an approximately normal distribution of the underlying outcome (SD ∼ IQR/1.35), and SDs were derived from 95% CIs using the corresponding standard errors and sample sizes. These conversions assume symmetry of the outcome distribution and comparable measurement scales across studies. Converted SDs were used solely to harmonize variance estimates for meta-analysis.

Prediction intervals were also computed to estimate the dispersion of musical treatment impacts in new study settings, accounting for both within- and between-study variance.

A common challenge in BD research is the frequent absence of reported SDs for change-from-baseline scores (Pearson and Smart, 2018). Following Cochrane guidelines (Higgins and Green, 2011), we imputed missing SDs using a conservative correlation coefficient of *r* = 0.8, consistent with assumptions adopted in previous comparable meta-analyses (Giuliano et al., 2017; Pearson and Smart, 2018).

To ensure consistency in the direction of treatment effects, outcome scores were harmonized by reversing the direction of specific scales when necessary. When studies used different instruments to assess the same outcome (e.g., UEF), scores were standardized or converted to a common metric to facilitate meaningful comparison. However, due to differences in scale types, scoring conventions, and units, some studies could not be included in joint subgroup analyses, limiting the comparability within certain subdomains. Nevertheless, the global pattern of results remained robust and informative.

Accordingly, separate meta-analyses were performed for each functional domain (e.g., UEF, gait, cognition), ensuring all effect sizes were aligned and interpretable. All statistical analyses were conducted using the META package in R (Balduzzi et al., 2019), and forest plots were used to visualize the distribution of effect sizes. The statistical significance of pooled effects was assessed using the z-test.

The review protocol of the present study has been registered in INPLASY (Registration number: INPLASY202610042; 10.37766/inplasy2026.1.004; inplasy.com).

## 3 Results

### 3.1 Bibliographic systematic review and meta-analysis

ABI refers to BD that occurs after birth due to external factors, such as TBI, or internal factors like stroke, aneurysm, tumor, infectious disease, or heart attack (non-TBI) (Maas et al., 2022). The diversity of brain injuries poses a significant challenge for music-based studies aiming to draw conclusive results regarding the scope and connections between music and these biological conditions. For instance, among the reviewed studies, we identified 15 articles focusing on TBI patients, 59 on non-TBI patients, and some targeting specific diagnoses like aphasia, alexia, agnosia, amusia, or Diffuse Brain Injury (DBI), Right Brain Damage (RBD), and Left-Brain Damage (LBD), or in ABI in general. Participants’ number varied considerably over the studies, with the majority involving small sample sizes and only a few including more than 50 participants. This diversity can be attributed to the inherent heterogeneity of the ABI population and the challenges of enrolling and managing large groups of patients with BD in musical activities. All identified studies fall within the fields of neuroscience and psychophysiology, comprising experimental research that investigates the restorative effects of music as a form of rehabilitation for individuals with BD (see Section 3.2). To extend this analysis, we further examined the types of MI used in these experimental studies. This approach allowed for a deeper exploration of the diverse applications of music as both a clinical tool and a subject of research in therapeutic contexts (see Section 3.3).

### 3.2 The therapeutic potential of music training in neurorehabilitation

The therapeutic potential of music training is well supported by scientific evidence, yet its clinical implementation remains limited. Since 2,000, a total of 90 studies have examined the effects of musical stimulation in individuals with BD, consistently reporting beneficial outcomes across a range of functions (Supplementary Table 1). However, considerable variability exists in study designs, participant profiles, and intervention protocols, which complicates standardization and broader clinical adoption.

The experimental designs of the included studies were heterogeneous. They comprised randomized controlled trials (RCTs), case-control studies, detailed case reports, studies employing neuroimaging to assess functional or structural brain changes, clinical trials, and quasi-experimental designs. Many of these investigations evaluated pre- and post-intervention effects or compared outcomes with alternative therapies or healthy control groups.

Analysis of this body of research reveals that music-based rehabilitation contributes to improvements in behavioral, cognitive, and motor functions, while also promoting neuroanatomical reorganization during recovery. These therapeutic effects are categorized by outcome domains such as motor function, communication skills, cognitive performance, emotional regulation, behavior, and social engagement. To strengthen the evidence base, we conducted a meta-analysis when sufficient data were available in these studies (*n* = 41), targeting specific outcome domains to provide a rigorous and quantitative synthesis of the findings. This approach enables a clearer and more systematic evaluation of music’s rehabilitative efficacy, underscoring its potential as a complementary tool in neurorehabilitation.

#### 3.2.1 Benefits of motor rehabilitation

A total of 43 studies have demonstrated the effectiveness of MI in motor rehabilitation for individuals with BD. Since 2,000, 14 studies have reported improvements in gait parameters, while 32 studies have focused on UEF recovery. In particular, Music-Supported Therapy (MST), a type of MI based on piano and percussion exercises, has gained recognition as a powerful tool for motor rehabilitation, with enhanced mobility emerging as the most consistently reported benefit across the reviewed studies (see also Supplementary Table 1).

##### 3.2.1.1 Gait functional recovery

Gait rehabilitation is crucial, as psychomotor impairments affecting walking patterns are a common consequence of cerebral damage. The results demonstrated significant improvements across all categories in participants who engaged in MI compared to control groups. Among the most widely used approaches, Rhythmic Auditory Stimulation (RAS) is particularly effective in gait training. RAS involves synchronizing movements with musical rhythms, often enhanced by a metronome to provide structured auditory cues (Cha et al., 2014; Hayden et al., 2009; Park et al., 2010; Sheridan et al., 2021; Thaut et al., 2007; Thompson et al., 2021). Studies that excluded musical stimuli from the RAS protocol were not included in this review. Notably, four comparative RCTs demonstrated that rhythmic stimulation therapies are more effective for gait rehabilitation than standard physiotherapy without music (Cha et al., 2014; Mazhari-Jensen et al., 2023; Park et al., 2010; Thaut et al., 2007). An innovative study, based on personalized rhythmic gait training which involves wearable sensors and a smartphone application with a user’s playlist, got to increase walking speed in stroke patients (Hutchinson et al., 2020).

A meta-analysis of 8 studies assessed gait-related outcomes, including general gait performance, step length, symmetry, cadence, balance, timing, and velocity (Figure 2). This meta-analysis offers robust evidence for the positive influence of MI on various aspects of gait performance in individuals with BD. The pooled analysis revealed a pooled MD of 3.76 (95%CI: 1.63–5.89) and a statistically significant overall effect (z-score = 3.46, *P* < 0.01), supporting the notion that musical stimulation can facilitate improvements in locomotor function. Nevertheless, the analysis revealed high heterogeneity across studies, with an *I*^2^ of 94% (τ^2^ = 23.87; *P* < 0.01), indicating marked variability in effect sizes. This level of heterogeneity is likely due to a range of methodological differences, including patient populations, intervention durations, outcome definitions, and measured outcomes (different assessment tools). Further reflecting this variability, the subgroup analysis found significant differences across the various gait outcomes assessed (χ^2^ = 22.08, df = 7, *P* < 0.01). When examining individual gait domains, several emerged as particularly responsive with great and statistically significant improvements. Gait general, gait length, gait cadence, and gait velocity demonstrated clear benefits from MI, with effect sizes that were both positive and meaningful. These outcomes showed particularly strong effects, as indicated by their respective subgroup *z*-values (1.51, 6.46, 1.84, and 2.19, respectively), all with *P* < 0.01, and relatively low heterogeneity (*I*^2^ values: 89% [τ^2^ = 6.51], 22% [τ^2^ < 0.01], 95% [τ^2^ = 73.56], and 98% [τ^2^ = 37.26]; respectively). These results suggest that MI may effectively support temporal and pacing aspects of walking. In addition, outcomes related to gait balance and symmetry displayed marginal significance, with subgroup *z*-values of 2.00 (*P* = 0.05) for gait balance general, 1.94 (*P* = 0.05) for gait balance timing, and CIs for the MD overlapping zero. This may indicate that while MI readily supports timing and stride regularity, more complex postural or symmetrical adjustments may require different or more targeted therapeutic strategies. Importantly, the overall prediction interval remained positive (MD: 3.76 [95%CI: 1.63–5.89]), suggesting that future studies are likely to observe beneficial effects even when accounting for between-study variability. The relatively low heterogeneity observed in some subgroups, such as gait balance timing and gait length, also strengthens the case for these specific metrics as reliable and responsive indicators of progress during music-based gait rehabilitation.

##### 3.2.1.2 Upper-extremity function (UEF) recovery

Twenty-six studies investigated the impact of musical interventions on UEF recovery, consistently reporting notable benefits. Research in this field suggests MI enhances motor function by engaging individuals in active instrumental training, such as playing the piano keyboard or electric drums, which provides direct feedback and sensory information. According to different authors, MI can improve: (*i*) Hand rehabilitation (Colombo et al., 2019; Kim et al., 2023; Raglio et al., 2017; Villeneuve and Lamontagne, 2013; Villeneuve et al., 2014), increasing finger movement velocity and pressing force (Bunketorp-Käll et al., 2017; Chong et al., 2014; Colombo et al., 2019; Friedman et al., 2011; Zondervan et al., 2016), (*ii*) Shoulder flexibility (Jeong and Kim, 2007; Peyre et al., 2023), (*iii*) Fine and gross motor skills of the hand and arm, including speed, precision, and smoothness. (Altenmüller et al., 2009; Nikmaram et al., 2019; Scholz et al., 2016; Van Vugt et al., 2014), (*iv*) Functional grasping and pinching movements (Segura et al., 2021), (*v*) Neuroplasticity and motor recovery, restoring connectivity between auditory and motor regions (Altenmüller et al., 2009; Amengual et al., 2013; Grau-Sánchez et al., 2013; Ripollés et al., 2016). In addition, Schneider et al. (2010) and Segura et al. (2024) demonstrated that MST is more efficient than conventional physiotherapy for the recovery of fine motor skills in stroke patients.

A comprehensive meta-analysis of 14 studies assessed the impact of MI on UEF across several outcome domains, including general motor performance, manual dexterity, hand strength, and shoulder mobility (Figure 3). The meta-analysis consolidates evidence from a broad range of studies assessing the effects of MI on UEF in patients with BD. The pooled analysis revealed a clear and statistically significant benefit, with a MD of 3.36 (95%CI: 2.28–4.44) and a highly significant test for overall effect (z-score = 6.11, *P* < 0.01). This strong signal indicates that, across diverse study designs and outcome measures, musical stimulation contributes positively to motor recovery in affected individuals. This strong effect was accompanied by a favorable prediction interval (−1.25; 7.97), reinforcing the likelihood that future studies will also detect improvements. Heterogeneity was moderate (*I*^2^ = 49%, τ^2^ = 4.92), reflecting methodological differences among studies, but subgroup differences were significant (χ^2^ = 40.77, df = 12, *P* < 0.01), suggesting that the effect size varied substantially across functional domains. This variation emphasizes the importance of considering specific functional domains when evaluating the impact of MI. Detailed subgroup analyses shed light on where the most robust effects occur. Seven of the 10 subgroups (for which there are statistical analyses) showed statistically significant subgroup effects, all with positive MDs; one additional subgroup demonstrated marginal statistical significance. Some of the most prominent and consistent benefits were observed in UEF general, manual dexterity, and shoulder flexibility, which are critical for functional independence in daily life. The BBT scores demonstrated a significant improvement with an MD of 4.41 (95%CI: 1.80–7.02; z-score = 3.31, *P* < 0.01) in UEF hand function/manual dexterity. Similarly, the Nine-Hole Pegboard Test (9HPT-pegs) scores showed a significant improvement of 1.15 (95%CI: 0.16–2.14; z-score = 2.29, *P* = 0.02). Both measures exhibited variable heterogeneity (BBT: *I*^2^ = 47%; 9HPT-pegs: *I*^2^ = 0%). These findings strongly suggest that rhythm and MI provide robust improvements in fine motor control and coordination. Task-oriented functional assessments also showed compelling effects. The ARAT produced one of the largest effect sizes (MD = 5.79, 95%CI: 2.79–8.79) with a strongly significant test (z-score = 3.78, *P* < 0.01), indicating substantial improvements in complex, goal-directed upper-limb tasks. Global impairment scales such as the Fugl-Meyer Assessment (FMA) showed a statistically significant improvement (z-score = 2.15, *P* = 0.03), reinforcing the idea that broader motor impairment measures are highly sensitive to short-term or specific functional changes induced by MI. Other outcomes in the general UEF, such as hand strength and shoulder flexibility, also demonstrated a strong effect, further supporting the conclusion that MI can markedly enhance task-specific motor recovery.

These findings underline the value of music-based protocols for promoting fine motor control and functional task performance, which are essential for independence in daily activities. The results also highlight a relative limitation in improving strength and joint flexibility, indicating that these domains may require complementary rehabilitation approaches. Particularly in these UEF outcomes, the observations suggest that future research should prioritize standardized protocols and explore the integration of music-based therapy with interventions targeting strength and proximal mobility to maximize overall recovery.

#### 3.2.2 Language and communication improvements

Twenty studies have reported that MI stimulate language recovery following BD. Fourteen of these studies were included in a meta-analysis, covering various language-related domains such as general communication, spontaneous speech, repetition, and naming. The findings consistently demonstrated greater improvements in participants receiving MI compared to control groups. Most of the studies focused on patients with aphasia, highlighting the effectiveness of musical treatments in enhancing communication abilities, particularly in several well-designed RCTs (Conklyn et al., 2012; Jungblut et al., 2022; Särkämö et al., 2008; Särkämö et al., 2014; Siponkoski et al., 2023; Ueda et al., 2024; van der Meulen et al., 2014). Reported improvements included reading and repetition abilities (Särkämö et al., 2008); articulation, prosody in spontaneous speech, naming, repetition, and comprehension (Jungblut et al., 2022); verbal memory and global language recovery (Sihvonen et al., 2020); expressive speaking and vocal range (Baker et al., 2005); as well as general communication and vocalization. Interestingly, neuroimaging studies have also shown increased connectivity in brain regions associated with language following MI (Bitan et al., 2018). Additionally, intensive singing has been found to enhance speech motor functions in individuals with non-fluent aphasia after BD (Marchina et al., 2023; Ueda et al., 2024).

A meta-analysis of these studies allows the evaluation of the effectiveness of rehabilitative interventions on communication outcomes in individuals with BD resulting from stroke or TBI (Figure 4). The pooled results indicate a statistically significant benefit of experimental interventions compared to control conditions, with a pooled MD of 3.24 (95%CI: 1.26–5.22) and a z-score of 3.21 (*P* < 0.01) in the test for overall effect. These findings suggest that these therapies contribute to meaningful improvements in language abilities. However, substantial heterogeneity was observed across studies (*I*^2^ = 73%, τ^2^ = 14.81, *P* < 0.01), likely reflecting variability in intervention types, outcome measures, and participant characteristics. Subgroup analyses revealed that certain communication domains responded more consistently to MI. In particular, outcomes related to communication repetition and naming (AAT) demonstrated robust and statistically significant improvements with high MD, such as repetition (MD = 8.85 and 95% of 4.75–12.95) and naming (MD = 7.68 and 95%CI of 3.79–11.56), with no observed heterogeneity (*I*^2^ = 0%, τ^2^ = 0, *P* > 0.60 for both) and strong effect sizes (z-score = 4.23 and z-score = 3.87, respectively; *P* < 0.01 for both). These findings highlight the responsiveness of these specific language functions to structured therapeutic input. General communication outcomes (e.g., ANELT, BDAE, AAT, TLC, SIS, CERAD/BNT) and spontaneous speech showed variable results, with several studies reporting wide CIs and non-significant effects but positive MD. This variability may be attributed to differences in assessment tools, sample sizes, and baseline impairment severity. The prediction interval for the overall effect (95%CI: −4.95 to 11.44) suggests that while future studies may often observe positive outcomes, negative or null effects remain possible, reinforcing the importance of considering patient-level factors, such as aphasia type and severity, when evaluating treatment efficacy.

The general pattern supports the clinical relevance of targeted speech and language MI, especially for repetition and naming abilities, and emphasizes the importance of integrating evidence-based approaches in the rehabilitation of acquired communication impairments following BD.

#### 3.2.3 Cognitive rehabilitation

Cognitive impairment is one of the most common consequences of BD (Loetscher et al., 2019; Murakami et al., 2014). Out of the 90 included studies in this review, 25 reported cognitive benefits following MI, with 11 focusing on memory, 9 on attention, and 12 on executive function.

Several studies have highlighted the role of music in memory recovery. One study demonstrated that listening to popular songs evoked autobiographical memories in five individuals with severe ABI, providing the first evidence of this effect in patients with severe BD (Baird and Samson, 2014). Other research has shown that listening to favorite music enhances brain connectivity and activates memory-related functions (Carrière et al., 2020; Särkämö et al., 2014). Moreover, different musical elements appear to influence memory recovery in distinct ways. For example, vocal music has been found to enhance verbal memory recovery more effectively than instrumental music or audiobooks (Sihvonen et al., 2020). Additionally, engaging in active musical activities, such as playing the piano, promotes cortical plasticity by stimulating neural connections, thereby improving memory, attention, and executive function in individuals with mild TBI (Jones et al., 2021; Vik et al., 2018).

Deficits in executive functions are considered core symptoms of TBI. Numerous studies suggest that MI effectively stimulates executive function, particularly through active musical engagement. Research has demonstrated that executive function is activated and improved through various musical experiences, including: (*i*) Playing musical instruments (Fujioka et al., 2018; Lynch and LaGasse, 2016; Martínez-Molina et al., 2021; Ripollés et al., 2016; Sihvonen et al., 2022a; Siponkoski et al., 2020; Vik et al., 2018), (*ii*) Singing therapy in aphasia recovery (Jungblut et al., 2022), and (*iii*) Musical improvisation (Thaut et al., 2009). A particularly compelling study by Sihvonen et al. (2022b) demonstrated that Neurological Music Therapy (NMT), which includes rhythmic training, cognitive-motor training, and piano and drum playing, induces structural white matter neuroplasticity in post-TBI patients, providing a biological basis for improved executive function.

Moreover, selective attention is significantly affected in ABI patients (Jeong et al., 2018). MST, which involves active training using electronic drums and a piano keyboard, has demonstrated significant improvements in attention, executive functions, information processing speed, and mental flexibility in individuals with chronic stroke (Fujioka et al., 2018; Ripollés et al., 2016). A recent study by Jeong et al. (2024) further demonstrated that virtual reality-based music attention training is an effective cognitive intervention for restoring attentional processes in the ABI population.

A meta-analysis on memory, attention, and executive function, incorporating 12 studies, confirms the positive impact of music on cognitive recovery in individuals with BD (Figure 5). The meta-analysis included data that allows assessing five key cognitive domains: working memory, verbal memory, visual memory, attention, and executive function. When all cognitive outcomes were pooled together, combining both executive function and general cognition, the analysis yielded a significant overall effect size of 0.89 (95%CI: 0.36−1.42) with strong statistical significance (z-score = 3.29, *P* < 0.01). Although some variability was observed between cognitive subgroups, the overall heterogeneity was relatively low (*I*^2^ = 40%, τ^2^ = 0.30, *P* = 0.02). The findings provide encouraging evidence for the efficacy of MI in enhancing cognitive outcomes in neurorehabilitation. However, the test for subgroup differences was statistically significant (χ^2^ = 36.32, df = 10, *P* = 0.01), suggesting that the magnitude of benefit might differ depending on the specific cognitive domain targeted. In the subgroup analysis, we found statistically significant results in four out of six subgroups with statistical measurements, all with positive MD and strong effects. In the general cognition subgroup, specifically verbal memory outcomes assessed using the RAVLT, the MD was 0.91 with CIs consistently showing a positive trend (95%CI: 0.30−1.52). The test for overall effect in this domain was statistically significant (z-score = 2.93, *P* < 0.01), indicating a small-to-moderate benefit of MI in cognitive performance. Importantly, heterogeneity within this subgroup was negligible (*I*^2^ = τ^2^ = 0%, *P* = 0.98), suggesting highly consistent results across studies. The visual memory subgroup measured using WAIS-III demonstrated a higher effect, with a pooled MD of 2.58 (95%CI: 0.89−4.28), with the overall subgroup result being statistically significant (z-score = 2.99, *P* < 0.01) and again no heterogeneity across studies (*I*^2^ = τ^2^ = 0%, *P* = 0.92). The results are even more robust in the executive function-TMT B subgroup, with a high and significant mean difference (MD = 14.42; 95%CI: 2.00−26.84) and strong effect (z-score = 2.28, *P* = 0.02) and no heterogeneity across studies (*I*^2^ = τ^2^ = 0%, *P* = 0.72), indicating a robust benefit of MI in executive function. This meta-analysis on cognitive rehabilitation provides compelling evidence that MI can significantly enhance cognitive recovery in individuals with BD, particularly in the domains of executive function, verbal memory, and visual memory. The general large effect size and consistent findings across studies (with minimal heterogeneity) highlight the reliability and robustness of these outcomes. While some domain-specific variability exists, the positive and statistically significant effects across key cognitive areas highlight the potential use of music as an effective tool in cognitive neurorehabilitation for this population.

#### 3.2.4 Emotional, behavioral, and social outcomes

Twenty-nine studies have reported improvements in emotional wellbeing following MI, while 25 studies have documented behavioral and social benefits, including better mood, regulated emotions, reduced agitation, enhanced consciousness, increased relaxation, and decreased symptoms of depression, among others (Cha et al., 2014; Ripollés et al., 2016; Fujioka et al., 2018; Jeong and Kim, 2007; Grau-Sánchez et al., 2013; Grau-Sánchez et al., 2018; Palumbo et al., 2022). A qualitative assessment of these studies provides deeper insight into music’s impact on behavioral and emotional regulation in patients with BD. Musical activities such as songwriting, singing, playing an instrument, choral singing, and improvisation were shown to enhance emotional wellbeing and mood stability in individuals with ABI (Baker et al., 2005; Baker et al., 2019; Jeong and Kim, 2007; Jun et al., 2013; Kim et al., 2011; Nayak et al., 2000; Park et al., 2016; Ripollés et al., 2016; Särkämö et al., 2008; Särkämö et al., 2014; Tamplin et al., 2013; Zumbansen et al., 2017).

Music’s ability to reduce agitation emerged as a key outcome in studies involving TBI patients (Bower et al., 2014; Park et al., 2016), as did its effect in reducing post-stroke depression (Palumbo et al., 2022). Furthermore, two neuroimaging studies used MRI scans to observe patients in vegetative or minimally conscious states after BD while they were exposed to music, revealing a positive effect on levels of consciousness (Carrière et al., 2020; Okumura et al., 2014). Another study involving six TBI patients employed EEG readings taken before, during, and after listening to a music raga, demonstrating that music can promote relaxation and deep sleep (Ushasree et al., 2021). A more recent study also demonstrated the efficacy of music listening in improving sleep during post-acute ABI rehabilitation (Palmquist et al., 2025). Additionally, a recent case report supported the use of music to reduce anxiety and promote relaxation, as confirmed through biofeedback measures in a TBI patient (Vaudreuil et al., 2024). Similarly, Ribeiro et al. (2014) found that music effectively induced relaxation in 13 individuals with severe cerebral damage in a vegetative state, and Fletcher et al. (2025) observed positive trends and reduced variability in anxiety and pain in acute stroke patients over 24 h. Several studies have confirmed that musical stimulation activates brain regions involved in emotional processing (Carrière et al., 2020; Sihvonen et al., 2020; Vik et al., 2018), providing neurobiological evidence of music’s role in emotional regulation and mood enhancement.

The social benefits of MI have been explored in a smaller number of studies, which reported improvements in social interaction and communication skills after the intervention (Jeong and Kim, 2007; Raglio et al., 2016; Seibert et al., 2000; Vik et al., 2018).

We conducted a meta-analysis to evaluate the effects of MI on emotional states in individuals with BD, synthesizing data across 13 emotional outcome domains, including depression, anxiety, emotional wellbeing, and self-care (Figure 6). The pooled analysis revealed a small but statistically significant overall effect favoring the intervention group (MD = 0.19; 95%CI: 0.03−0.35), with a z-score of 2.35 (*P* = 0.02). Moderate heterogeneity was observed (*I*^2^ = 52%, τ^2^ = 0, *P* < 0.01), indicating variability in effect sizes across studies. The test for subgroup differences across outcome domains (e.g., depression, pain, anxiety, emotional wellbeing, and self-care) was not statistically significant (χ^2^ = 14.49, df = 12, *P* = 0.27), suggesting that no single domain demonstrated a consistently stronger effect than others.

Within the depression-related outcomes, results were mixed. The PHQ-9 and BDI-II indicated positive effects, whereas the CES-D yielded a small negative effect (MD = −1.10; 95%CI: −5.58 to 3.38), and the 95%CIs for anxiety burden and pain included zero, indicating non-significance.

For emotional wellbeing and depression, the study by Palumbo et al. (2022) showed a meaningful and statistically significant improvement (MD = 3.44, 95%CI: 0.19–6.69; MD = 4.35, 95%CI: 1.10–7.60; respectively). Stroke Impact Scale Emotions (SIS-Emotions) scores also showed a positive pooled effect (MD = 4.82; 95%CI: −2.48−12.11), although with high heterogeneity (*I*^2^ = 78%, τ^2^ = 31.34, *P* = 0.01) and a non-significant test for subgroup effect (z-score = 1.29, *P* = 0.20). Self-care domains, assessed through the Strategies Used by People to Promote Health (SUPPH) subscales, consistently showed positive MD, but most had wide confidence intervals that crossed zero, indicating statistical non-significance.

Despite variability across individual outcomes, the results support the potential of MI to enhance emotional wellbeing and related domains in individuals with BD. However, the presence of moderate to high heterogeneity in several subgroups highlights the need for cautious interpretation and suggests that further research is needed to clarify which domains and populations benefit most.

#### 3.2.5 Quality of life

Notably, some studies identified enhanced QoL as a direct outcome of MI (Cha et al., 2014; Fujioka et al., 2018; Grau-Sánchez et al., 2013; Grau-Sánchez et al., 2018; Jeong and Kim, 2007; Palumbo et al., 2022; Ripollés et al., 2016), including many global outcomes such as psychological wellbeing, physical recovery, or social engagement.

We carried out a meta-analysis to evaluate the impact of MI on QoL in individuals with BD, drawing on data from five studies and three standardized QoL measures (Figure 7): the Stroke-Specific Quality of Life Scale (SS-QoL), the Quality of Life after Brain Injury (QoLIBRI), and the Quality-of-Life Index (QLI). The pooled findings offer a cautiously optimistic view, though overall results did not reach statistical significance (z-score = 1.45; *P* = 0.15). The overall effect size favored the music intervention group, with a pooled MD of 4.44 (95%CI: −1.56 to 10.43). The overall heterogeneity across studies was substantial (*I*^2^ = 73%, τ^2^ = 34.06, *P* < 0.01), indicating variability in effect estimates. The test for subgroup differences between the three QoL measures (SS-QoL, QoLIBRI, and QLI) was not significant (χ^2^ = 1.56, df = 2, *P* = 0.46), suggesting that neither measure consistently outperformed the other in capturing treatment effects. In the SS-QoL subgroup, three studies were included. Within the SS-QoL subgroup, Cha et al. (Cha et al., 2014) reported a large and significant benefit (MD = 18.90, 95%CI: 8.40–29.40), while Jeong and Kim (2007) reported a negligible and non-significant effect (MD = −0.05, 95%CI: −0.46–0.36), and Grau-Sánchez et al. (2018) indicated a moderate but non-significant improvement (MD = 7.40, 95%CI: −5.62–20.42). The pooled estimate from this subgroup yielded an MD of 7.86 (95%CI: −3.66–19.38), indicating a trend toward benefit but lacking statistical significance (z-score = 1.34, *P* = 0.18). Notably, heterogeneity in this subgroup was substantial (*I*^2^ = 85%, τ^2^ = 82.72, *P* < 0.01), suggesting that differences in study design or sample characteristics may explain the variation in observed effects. For the QoLIBRI subgroup, the analysis included a single study (Sihvonen et al., 2022b), which reported a small, non-significant improvement in QoL (MD = 2.80, 95%CI: −4.33 to 9.93). Finally, the QLI subgroup, represented by Palumbo et al. (2022), also found a minimal and non-significant effect (MD = 0.74, 95%CI: −2.10 to 3.58). The lack of additional studies in these subgroups limits broader generalization, but it contributes to the overall model assessing QoL effects across both tools. Overall, the meta-analysis suggests a potential benefit of MI on QoL in individuals with BD, though current evidence remains inconclusive due to limited studies and high heterogeneity.

#### 3.2.6 Global improvement

The outcome of global improvement has recently been considered an important variable in studies investigating music-based rehabilitation for ABI (Jeong et al., 2024; Kim et al., 2023; Palumbo et al., 2022). However, previous reviews did not consider it as a measure of global improvement (Magee et al., 2017). The variability in the concept of global improvement is evident, as some measures focus on comprehensive cognitive assessments, such as the Clinical Dementia Rating (CDR) or the Global Deterioration Scale (GDS) (Jeong et al., 2024), while others assess the level of disability in daily activities, such as the Functional Independence Measure (FIM) (Kim et al., 2023). Palumbo et al. (2022) evaluated global post-stroke disability through clinical interviews using the modified Rankin Scale (mRS). However, the SIS, a self-reported instrument, is widely recognized for complementing the understanding of global recovery and improvement (Palumbo et al., 2022; Segura et al., 2024).

The meta-analysis on global improvement outcomes (Figure 8) across five studies found no statistically significant overall effect of MI in individuals with ABI (MD = 0.03, 95%CI:−0.34 to 0.41, z-score = 0.18, *P* = 0.86), with moderate but non-significant heterogeneity (*I*^2^ = 31%, τ^2^ = 0.04, *P* = 0.20). Subgroup analysis by outcome measure (CDR-SB, GDS, FIM, mRS, and SIS Recovery) revealed no significant differences (χ^2^ = 6.61, df = 4, *P* = 0.16), though the SIS Recovery subgroup showed a positive trend with marginal statistical significance (z-score = 1.81, *P* = 0.07) and no heterogeneity (*I*^2^ = τ^2^ = 0%). Current evidence does not support a significant global improvement effect from MI on ABI populations across the included measures. However, the SIS Recovery domain showed a promising trend that warrants further investigation, particularly in larger, more targeted studies.

#### 3.2.7 Sensitivity analysis

We conducted a comparative meta-analytic assessment using (*i*) the complete set of eligible studies and (*ii*) a restricted dataset excluding studies identified as outliers, to evaluate whether the overall effect estimates for the main outcome categories and their respective subcategories retained statistical significance. Studies flagged as outliers (*n* = 15) are marked with an asterisk in Figures 2–8. A summary of these comparisons is provided in Table 1.

Comparisons were performed only for overall categories and subcategories that included at least two studies in both analytical scenarios. As shown in Table 1, outlier studies were identified exclusively within the outcomes: gait, UEF, and cognitive domains, encompassing several subcategories within these three outcome groups. Importantly, across all evaluated categories and subcategories, the statistical significance observed in the primary meta-analysis was preserved after exclusion of the outlier studies, indicating the robustness of the overall findings.

Notably, for 9 of the 16 outcomes and sub-outcomes evaluated in the sensitivity analysis, exclusion of outlier studies resulted in stronger effect sizes, with eight of these also showing increased z-score values (Table 1). Measures of heterogeneity (*I*^2^ and τ^2^) were generally comparable between analyses, although in a small number of cases these metrics showed substantial increases or decreases (Table 1).

### 3.3 Types of musical interventions in brain damage studies

This review examines the types of musical stimulation used in MI for ABI, most of which focus on rehabilitation, intending to link specific interventions to reported outcomes across 90 studies (section 3.2). A major challenge in assessing the efficacy of MI lies in the substantial variability in their duration, ranging from under an hour to, in rare cases, over 60 h (Table 2). Remarkably, only 11 studies implemented long-term interventions (≥ 30 h), and these were consistently associated with significant improvements across multiple domains. These included: (i) MST (Altenmüller et al., 2009; Fujioka et al., 2018; Segura et al., 2021), (*ii*) Rhythm- and music-based therapy (Bunketorp-Käll et al., 2017), (*iii*) Choral and singing therapy (Tamplin et al., 2013), and (*iv*) Music listening interventions (Särkämö et al., 2008; Sihvonen et al., 2020). Active music interventions, where participants engage directly with music through instrument playing or singing, were the most prevalent among these high-impact studies (Table 2).

**TABLE 2:** Types of music-based interventions (MI) in studies on brain damage (BD).

Reference	Musical intervention	TE	Benefits
**Interventions involving musical instrument playing**			
Chong et al. (2014)	Piano or keyboard playing	5	U
Bernardi et al. (2017)	Piano or keyboard playing	2	BS/CF/U
Vik et al. (2018)	Piano or keyboard playing	4	A/BS/CF/EF/ME/EW
Villeneuve and Lamontagne (2013)	Piano or keyboard playing	9	U
Villeneuve et al. (2014)	Piano or keyboard playing	9	U
Kim et al. (2023)	Piano or keyboard playing	4.5	U
Altenmüller et al. (2009)	MST	7.5	U
Schneider et al. (2007)	MST	7.5	U
Schneider et al. (2010)	MST	7.5	U
Amengual et al. (2013)	MST	10	U
Grau-Sánchez et al. (2013)	MST	10	BS/U
Tong et al. (2015)	MST	10	U
Ripollés et al. (2016)	MST	10	A/BS/CF/EF/EW/U
Grau-Sánchez et al. (2018)	MST	10	BS/C/EW
Rojo et al. (2011)	MST	20	G/U
Van Vugt et al. (2014)	MST	5	EW/U
Fujioka et al. (2018)	MST	30	C/BS/CF/EF/EW/U
Grau-Sánchez et al. (2017)	MST	18	U
Segura et al. (2021)	MST	30	U
Segura et al. (2024)	e-MST	40	C/U/CF
Fujioka et al. (2025)	MST	30	CF
Martínez-Molina et al. (2021)	NMT	20	CF/EF
Lynch and LaGasse (2016)	NMT	6	CF/EF
Sihvonen et al. (2022b)	NMT	24	CF/EF
Street et al. (2018)	NMT	6	U
Siponkoski et al. (2020)	NMT	24	BS/CF/EF
Siponkoski et al. (2022)	NMT	24	C/BS/EW
Vaudreuil et al. (2024)	NMT	8	EW
Raghavan et al. (2016)	MULT-I	9	EW/U
Palumbo et al. (2022)	MULT-I	9	EW/U
Raglio et al. (2017)	RAMT	10	G/EW/U
Jeong et al. (2024)	VR-MAT	20	A/EF
**Interventions combining music and movement**			
Thompson et al. (2021)	RAS	6	G
Jeong and Kim (2007)	RAS	16	BS/EW/U
Sheridan et al. (2021)	RAS	4.5	G
Thaut et al. (2007)	RAS	15	BS/G
Cha et al. (2014)	RAS	7.5	BS/G
Suh et al. (2014)	RAS	4.5	G
Elsner et al. (2020)	RAS	6	G
Hayden et al. (2009)	RAS	**	G
Park et al. (2010)	FTAS	10	G
Bunketorp-Käll et al. (2017)	RMT	36	G/ME/U
Mazhari-Jensen et al. (2023)	An exercise program while listening to music	1.5	G
Friedman et al. (2011)	MusicGlove therapy (hand movements)	< 1	U
Zondervan et al. (2016)	MusicGlove therapy (hand movements)	<1	U
Scholz et al. (2016)	Sonification	5	U
Scholz et al. (2015)	Sonification		U
Colombo et al. (2019)	Sonification	< 1	U
Nikmaram et al. (2019)	Sonification	15	U
Raglio et al. (2021)	Sonification	7.5	U/BS/EW
Peyre et al. (2023)	Sonification	3	U
Schauer and Mauritz (2003)	Walking while listening to music	5	G
Wang et al. (2021)	Walking while listening to music	24	G
Hutchinson et al. (2020)	Music-Based digital therapeutic	< 1	G
**Interventions based on singing**			
Tamplin et al. (2013)	Choir	40	BS/C/EW
Zumbansen et al. (2017)	Choir	34	C/EW
Conklyn et al. (2012)	Modified-MIT	< 1	C
Bitan et al. (2018)	MIT	24	C
van der Meulen et al. (2014)	Intensive-MIT	30	C
Siponkoski et al. (2022)	MIT	48	C
Ueda et al. (2024)	MIT	3.3	C
Marchina et al. (2023)	MIT	112.5	C
Jungblut et al. (2022)	Voice training SIPARI	24	BS/C/CF/EF
Baker et al. (2005)	Favorite song***	12.5	C/EW
Bower et al. (2014)	Favorite song***	2.5	BS
**Interventions involving passive music listening**			
Fan et al. (2024)	Personalized playlist	90	CF/EW
Ribeiro et al. (2014)	Relaxing music****	6	BS
Park et al. (2016)	Classical relaxing music/preferred music	9	BS/EW
Kasdan and Kiran (2018)	Musical exercise	1	CF/C
Särkämö et al. (2008)	Favorite music	60	A/C/CF/ME/EW
Särkämö et al. (2014)	Favorite music	60	A/C/CF/ME/EW
Carrière et al. (2020)	Favorite music	< 1	BS/C/CF/ME/EW
Särkämö et al. (2010)	Favorite music	≥ 60	ME
Sihvonen et al. (2020)	Popular/jazz/classical and film music	60	C/CF/ME/EW
Ushasree et al. (2021)	Traditional songs (indian ragas)	< 1	BS
Baird and Samson (2014)	Popular songs	*	CF/ME
Okumura et al. (2014)	3 clips: beat/instrumental/vocal	0.03	BS
Fletcher et al. (2025)	Popular/jazz/classical/relaxing	< 6	P, EW
Palmquist et al. (2025)	Relaxing music (classical, jazz, new age)	5	P, EW
Aravantinou-Fatorou and Fotakopoulos (2021)	Traditional songs (Greek songs)	72	C
Baylan et al. (2020)	Classical/pop/rock	56	C, ME, EW, A
**Other active and combining musical activities**			
Jones et al. (2021)	MACT listening and playing	3	A/CF
Baker et al. (2019)	Therapeutic songwriting	12	EW
Seibert et al. (2000)	Listening and playing	**	A/BS/C/CF/EF/M/EW
Nayak et al. (2000)	Mixed music therapy techniques	10	BS/EW
Thaut et al. (2009)	Singing and playing improvised music	2	CF/EF/EW
Jun et al. (2013)	Music movement therapy (singing and playing)	24	U/EW
Raglio et al. (2016)	Active Music therapy based on improvisation	22.5	C
Kim et al. (2011)	Singing, playing, and listening	5.5	EW

Abbreviations for MI are as follows, FTAS, Fast-tempo auditory stimulation; MACT, Music Attention Control Training; MIT, Melodic Intonation Therapy; MULT-I, Music Upper Limb Therapy-Integrated; NMT, Neurological Music Therapy; MST, Music-supported Therapy; e-MST, Enriched-Music-supported Therapy; RAMT, Relational Active Music Therapy; RAS, Rhythmic Auditory Stimulation; RMT, Rhythm- and Music-Based Therapy; SIPARI, Singing, Intonation, Prosody, Breathing, Rhythm, Improvisation; VR-MAT, Virtual Reality-based Music Attention; TE, Time Exposure in Hours. Abbreviations for benefits, A, Attention; BS, Behavioral and Social; C, Communication; CF, Cognitive Functions; EF, Executive Function; G, Gait; ME, Memory; EW, Emotional Wellbeing; U, Upper Extremity Function; P, Pain. (*: unclear, one session is reported; **: not specified; ***: with guitar accompaniment; ****: Radio/instrumental classical music/instrumental relaxing music with nature sounds).

#### 3.3.1 Interventions involving musical instrument playing

In 32 studies, active music training, such as playing a musical instrument, was a fundamental component of restorative therapy targeting both fine and gross motor upper-limb skills. Among these, 22 studies reported significant improvements in UEF in individuals with BD. Piano playing, in particular, has been highlighted as an effective intervention for hand rehabilitation (Chong et al., 2014; Kim et al., 2023; Villeneuve and Lamontagne, 2013; Villeneuve et al., 2014) as well as for cognitive enhancement in executive functions such as attention, learning strategies, and memory retrieval (Bernardi et al., 2017; Vik et al., 2018). Grau-Sánchez et al. (2017) reported notable improvements in keyboard task performance after just one session of piano playing, while Segura et al. (2024) confirmed enhanced motor recovery through enriched MST compared to a conventional motor program.

MT techniques involving instrumental playing, such as MST, which typically includes playing electronic instruments such as a MIDI keyboard and a drum set, was the most frequently applied approach, reported in 15 studies (Altenmüller et al., 2009; Amengual et al., 2013; Fujioka et al., 2018; Fujioka et al., 2025; Grau-Sánchez et al., 2013; Grau-Sánchez et al., 2017; Grau-Sánchez et al., 2018; Ripollés et al., 2016; Rojo et al., 2011; Schneider et al., 2007; Schneider et al., 2010; Segura et al., 2021; Segura et al., 2024; Tong et al., 2015; Van Vugt et al., 2014). Additionally, NMT demonstrated the rehabilitative potential of active MI in seven studies (Lynch and LaGasse, 2016; Martínez-Molina et al., 2021; Sihvonen et al., 2022a; Siponkoski et al., 2020; Siponkoski et al., 2022; Street et al., 2018; Vaudreuil et al., 2024).

#### 3.3.2 Interventions combining music and movement

Restoring walking ability is a key milestone in cerebral damage rehabilitation, and MI involving movement synchronized with music has demonstrated efficacy in motor recovery, which is proven among the 22 included studies (Table 2). Thirteen studies focusing on rhythmic stimulation reported significant gait improvements (Bunketorp-Käll et al., 2017; Bunketorp-Käll et al., 2017; Cha et al., 2014; Park et al., 2010; Schauer and Mauritz, 2003; Sheridan et al., 2021; Thaut et al., 2007; Thompson et al., 2021; Wang et al., 2021). Among these, Rhythmic Auditory Stimulation (RAS) and Fast Tempo Auditory Stimulation (FTAS), both of which typically incorporate a metronome and sometimes preferred music, were the most used techniques. Also, 10 studies reported an improvement in UEF thanks to interventions based on music and movement. Novel music-based rehabilitation approaches, Sonification of arm and hand movements and MusicGlove therapy have also been explored (Nikmaram et al., 2019; Peyre et al., 2023; Raglio et al., 2021; Scholz et al., 2015; Scholz et al., 2016; Zondervan et al., 2016).

#### 3.3.3 Interventions based on singing

Singing therapy has played a prominent role in 11 studies, with interventions including: (*i*) Choir participation (Tamplin et al., 2013; Zumbansen et al., 2017), (*ii*) Melodic Intonation Therapy (MIT) exercises (Bitan et al., 2018; Conklyn et al., 2012; Marchina et al., 2023; Siponkoski et al., 2023; Ueda et al., 2024; van der Meulen et al., 2014), (*iii*) Singing favorite songs with guitar accompaniment (Baker et al., 2005; Bower et al., 2014), and (*iv*) Intensive voice training, incorporating intonation, prosody, breathing, rhythm, and improvisation (Jungblut et al., 2022). The primary benefit of singing-based interventions is enhanced communication abilities in aphasic patients, with multiple studies demonstrating that singing is more effective than conventional language rehabilitation therapies (Conklyn et al., 2012; Jungblut et al., 2022; Siponkoski et al., 2023; Ueda et al., 2024; van der Meulen et al., 2014).

#### 3.3.4 Interventions involving passive music listening

Music listening, as a passive intervention in which participants are exposed to recorded music, has been examined in 16 experimental studies employing various musical genres and styles, including: (*i*) popular songs (Baird and Samson, 2014; Ushasree et al., 2021), (*ii*) favorite music of participants (Aravantinou-Fatorou and Fotakopoulos, 2021; Carrière et al., 2020; Fan et al., 2024; Särkämö et al., 2008; Särkämö et al., 2010; Särkämö et al., 2014), and (*iii*) comparisons between different types of music (Baylan et al., 2020; Okumura et al., 2014; Park et al., 2016; Ribeiro et al., 2014), such as vocal *vs.* instrumental (Sihvonen et al., 2020) or classical vs. preferred music (Park et al., 2016) and (*iv*) relaxing music (Palmquist et al., 2025; Ribeiro et al., 2014). The main benefits of music listening were emotional wellbeing, reported in nine studies, and cognitive function, reported in seven studies (Table 2), with five of those focusing on memory enhancement. Sihvonen et al. (2020) suggested that listening to vocal music is an effective, easily applicable tool for language and cognitive recovery after a stroke. Additionally, communication and behavioral improvements were reported in 10 studies, further demonstrating the diverse benefits of this low-demand intervention.

## 4 Discussion

This systematic review and meta-analysis examined the therapeutic impact of MI on the rehabilitation of individuals with BD, revealing consistent evidence that musical engagement supports recovery across multiple domains, both physiological and psychological.

Motor impairments, which are among the most prevalent consequences of BD, showed notable improvements when treated with music-based therapies. In particular, gains in mobility, including gait rehabilitation and UEF, were among the most frequently reported outcomes. These improvements appear to be mediated by mechanisms such as rhythmic entrainment, auditory-motor coupling, and reward-emotional mediating mechanisms that might facilitate motor timing, coordination, and motivation during therapy (see for a review; Grau-Sánchez et al., 2020).

Beyond motor domains, communication outcomes also demonstrated favorable effects in several studies. MI, particularly through structured rhythmic and melodic interventions such as singing or MIT, has been associated with improved language production and comprehension, especially in individuals with aphasia (Marchina et al., 2023; Siponkoski et al., 2023). These interventions may promote functional reorganization in affected neural circuits and strengthen connectivity in perilesional regions (Jungblut et al., 2022; Särkämö et al., 2014; Sihvonen et al., 2020). However, the current evidence base is constrained by the limited number of studies, short intervention durations, and significant methodological heterogeneity, emphasizing the need for further research with more rigorous designs and larger sample sizes.

Cognitive domains showed particularly strong and consistent effects, with improvements observed in memory, executive function, and attentional control. Interventions involving music listening, rhythmic movement, and active music-making appear to contribute not only to behavioral gains but also to neuroplastic changes detectable through neuroimaging. These include structural and functional adaptations in frontal and temporal regions associated with higher-order cognitive processing (Särkämö et al., 2014; Sihvonen et al., 2020; Siponkoski et al., 2020). The converging evidence from behavioral and imaging studies reinforces the role of music as a potent multisensory stimulus capable of driving cognitive recovery. Additionally, behavioral and psychosocial outcomes, including mood, relaxation, consciousness, and social interaction, were reported in numerous studies. However, findings in these domains were more variable, particularly regarding QoL, which, despite showing directionally positive trends, did not achieve statistical significance. This likely reflects the small sample sizes, variability in outcome measures, and subjective nature of QoL assessments.

MI demonstrated small but statistically significant improvements in emotional outcomes, particularly in emotional wellbeing and selected domains of depression and anxiety. While behavioral outcomes, such as self-care practices measured through SUPPH subscales (e.g., coping, stress reduction, and decision-making), showed consistently positive trends, most effects did not reach statistical significance (Palumbo et al., 2022; Segura et al., 2024). Social outcomes, though less directly assessed, were indirectly reflected in improvements in emotional expression (e.g., via the SIS-Emotions subscale), suggesting potential benefits for interpersonal engagement. Collectively, these findings highlight music’s capacity to support emotional regulation and foster behaviors conducive to psychosocial functioning in individuals with BD.

An important dimension addressed in this review is the impact of BD on musical abilities themselves. While acquired amusia is a common impairment following BD (Sihvonen et al., 2017b), several studies also highlight a surprising preservation of certain musical functions, even in the presence of significant injury. Notably, individuals with prior musical training often demonstrate resilience to neurological damage, suggesting a potential neuroprotective effect of long-term musical engagement through the reinforcement of auditory, motor, and emotional neural networks. This dual pattern of vulnerability and resilience, manifested in disrupted rhythm and emotional perception on one hand, and preserved musical memory and pleasure on the other, highlights the complex ways in which BD affects musical processing. Lifelong engagement with music may thus play a modulatory role in buffering against cognitive and sensory decline (Belfi and Tranel, 2014).

Taken together, the findings of our meta-analysis offer robust support for the clinical utility of MI in neurorehabilitation, as seen previously in Alzherimer (Navarro et al., 2023) and ASD (Navarro et al., 2025). Improvements were consistently observed across motor, cognitive, and, albeit more variably, communicative and psychosocial domains. These outcomes emphasize the promise of music as an accessible, engaging, and cost-effective adjunct to conventional rehabilitation approaches.

Nevertheless, several limitations must be acknowledged. The high heterogeneity in study parameters, including differences in BD types, intervention modalities, and treatment durations, complicates cross-study comparisons and limits the generalizability of results. Moreover, music is an inherently complex and multifaceted form of art that integrates multiple sound parameters (e.g., intensity, velocity, pitch, timbre), compositional techniques, and genres. As a therapeutic intervention, music encompasses a wide variety of activities and stimuli, making precise categorization and systematic analysis challenging. In particular, the lack of detailed and standardized reporting of musical parameters (such as tempo, genre, instrumentation, mode, intensity, or presence of lyrics) across primary studies limits replicability and hampers the identification of specific musical elements underlying the observed effects. Furthermore, inconsistencies in neuropsychological assessment tools and a lack of standardized outcome measures reduce the reliability of meta-analytical synthesis. This methodological heterogeneity also constrained the feasibility of conducting subgroup and sensitivity analyses based on intervention type, etiology, duration, or musical characteristics. In many instances, the number of studies within potential subgroups was insufficient to support statistically robust stratified analyses, thereby limiting more granular exploration of sources of heterogeneity. Sensitivity analyses were therefore restricted and could only be applied selectively to assess the robustness of pooled findings following the exclusion of outlier studies. Statistical challenges stemming from non-uniform methodologies and small sample sizes further emphasize the need for more rigorous, large-scale trials employing standardized protocols. Despite these limitations, the global body of evidence affirms the therapeutic potential of MI and supports its integration into interdisciplinary neurorehabilitation programs aimed at optimizing recovery in individuals with neurological impairment.

To sum up, this study highlights the multifaceted potential of MI in the rehabilitation of individuals with BD, demonstrating consistent benefits across motor, cognitive, and, to a lesser extent, communicative and psychosocial domains. Moreover, the review expands understanding of music’s capacity to support emotional regulation, self-care behaviors, and social connectedness in this population. To further establish MI as a validated rehabilitation approach, future research should prioritize longitudinal studies with extended intervention duration to assess sustained outcomes, larger samples to strengthen statistical power, and standardized neuropsychological and biomarker evaluations, including transcriptomic and epigenomic profiling, to enhance cross-study comparability. Addressing these methodological challenges will be crucial to fully elucidate the mechanisms through which music promotes neural plasticity and recovery, and to support its systematic integration into evidence-based neurorehabilitation programs for individuals living with the consequences of BD.

## Sensogenomics working group

Antonio Salas Ellacuriaga —PI; Federico Martinón-Torres—PI; Laura Navarro Ramón—Coordinator.

### GenPoB/GenVip—Instituto de Investigación Sanitaria (IDIS) (alphabetic order)

Alba Camino Mera, Albert Padín Villar, Alberto Gómez Carballa, Alejandro Pérez López, Alicia Carballal Fernández, Ana Cotovad Bellas, Ana Isabel Dacosta Urbieta, Narmeen Mallah, Ana María Pastoriza Mourelle, Ana María Senín Ferreiro, Andrés Muy Pérez, Antía Rivas Oural, Antonio Justicia Grande, Antonio Piñeiro García, Anxela Cristina Delgado García, Belén Mosquera Pérez, Blanca Díaz Esteban, Carlos Durán Suárez, Carmen Curros Novo, Carmen Gómez Vieites, Carmen Rodríguez-Tenreiro Sánchez, Celia Varela Pájaro, Claudia Navarro Gonzalo, Cristina Serén Trasorras, Cristina Talavero González, Einés Monteagudo Vilavedra, Estefanía Rey Campos, Esther Montero Campos, Fernando Álvez González, Fernando Caamaño Viñas, Francisco García Iglesias, Gloria Viz Rodríguez, Hugo Alberto Tovar Velasco, Irene Álvarez Rodríguez, Irene García Zuazola, Irene Rivero Calle, Iria Afonso Carrasco, Isabel Ferreirós Vidal, Isabel Lista García, Isabel Rego Lijo, Iván Prieto Gómez, Iván Quintana Cepedal, Jacobo Pardo Seco, Jesús Eirís Puñal, José Gómez Rial, José Manuel Fernández García, José María Martinón Martínez, Julia Cela Mosquera, Julia García Currás, Julián Montoto Louzao, Lara Martínez Martínez, Laura Navarro Ramón, Lidia Piñeiro Rodríguez, Lorenzo Redondo Collazo, Lúa Castelo Martínez, Lucía Company Arciniegas, Luis Crego Rodríguez, Luisa García Vicente, Manuel Vázquez Donsión, María Dolores Martínez García, María Elena Gamborino Caramés, María Elena Sobrino Fernández, María José Currás Tuala, María Martínez Leis, María Soledad Vilas Iglesias, María Sol Rodriguez Calvo, María Teresa Autran García, Marina Casas Pérez, Marta Aldonza Torres, Marta Bouzón Alejandro, Marta Lendoiro Fuentes, Miriam Ben García, Miriam Cebey López, Montserrat López Franco, Nour El Zahraa Mallah, Narmeen Mallah, Natalia García Sánchez, Natalia Vieito Perez, Patricia Regueiro Casuso, Ricardo Suárez Camacho, Rita García Fernández, Rita Varela Estévez, Rosaura Picáns Leis, Ruth Barral Arca, Sandra Carnota Antonio, Sandra Viz Lasheras, Sara Pischedda, Sara Rey Vázquez, Sonia Marcos Alonso, Sonia Serén Fernández, Susana Rey García, Vanesa Álvarez Iglesias, Victoria Redondo Cervantes, Vanesa Álvarez Iglesias, Wiktor Dominik Nowak, Xabier Bello Paderne, Xabier Mazaira López.

### Nursing volunteers (alphabetic order)

Alejandra Fernández Méndez, Ana Isabel Abadín Campaña, Ana María León Caamaño, Ana María Buide Illobre, Ángeles Mera Cores, Carmen Nieves Vastro, Carolina Suarez Crego, Concepción Rey Iglesias, Cristina Candal Regueira, Dolores Barreiro Puente, Elvira Rodríguez Rodríguez, Eugenia González Budiño, Eva Rey Álvarez, Fernando Rodríguez Gerpe, Gemma Albela Silva, Isabel Castro Pérez, Isabel Domínguez Ríos, José Ángel Fernández de la Iglesia, José Cruces Vázquez, José Luis Cambeiro Quintela, José Ramón Magariños Iglesias, Julia Rey Brandariz, Julio Abel Fernández López, Luisa García Vicente, Manuel González Lito, Manuel González Lijó, Manuela Pérez Rivas, Margarita Turnes Paredes, María Aurora Méndez López, María Begoña Tomé Arufe, María Campos Torres, María del Carmen Baloira Nogueira, María del Carmen García juan, María Esther Moricosa García, María Luz Chao Jarel, María Martínez Leis, María Mercedes Jiménez Santos, María Salomé Buide Illobre, María Victoria López Pereira, Mercedes Jorge González, Mercedes Isolina Rodríguez Rodríguez, Miren Payo Puente, Natalia Carter Domínguez, Olga María Reyes González, Pilar Mera Rodríguez, Purificación Sebio Brandariz, Salomé Quintáns lago, Yolanda Rodríguez Taboada, María Pereira Grau.

### Other volunteers (alphabetic order)

Alba Arias Gómez, Alejandro Moreno Díaz, Ana Arca Marán, Astro González Guirado, Brais García Iglesias, Carlos Sánchez Rubín, Carmen Otero de Andrés, Clara Pérez Errazquin Barrera, Claudia Rey Posse, Cristina Rojas García, Eduardo Xavier Giménez Bargiela, Elena Gloria Morales García, Fabio Izquierdo García Escribano, Gabriel Guisande García, Jaime López Martín, Lara Pais Ramiro, Lucía Rico Montero, Luís Estévez Martínez, Manuel Estévez Casal, María Aránzazu Palomino Caño, María Rubio Valdés, Marisol Nogales Benítez, Miryam Tilve Pérez, Nuria Villar Muiños, Pablo Del Cerro Rodríguez, Pablo Pozuelo Martínez Cardeñoso, Salma Ouahabi El Ouahabi, Santiago Vázquez Calvache.

## Footnotes

1
https://www.bmj.com/content/372/bmj.n71

## References

[B1] Altenmüller E. Marco-Pallares J. Münte T. Schneider S. (2009). Neural reorganization underlies improvement in stroke-induced motor dysfunction by music-supported therapy. *Ann. N. Y. Acad. Sci.* 1169 395–405. 10.1111/j.1749-6632.2009.04580.x 19673814

[B2] Amengual J. Rojo N. Veciana de Las Heras M. Marco-Pallarés J. Grau-Sánchez J. Schneider S. (2013). Sensorimotor plasticity after music-supported therapy in chronic stroke patients revealed by transcranial magnetic stimulation. *PLoS One* 8:e61883. 10.1371/journal.pone.0061883 23613966 PMC3629163

[B3] Aravantinou-Fatorou K. Fotakopoulos G. (2021). Efficacy of exercise rehabilitation program accompanied by experiential music for recovery of aphasia in single cerebrovascular accidents: A randomized controlled trial. *Ir. J. Med. Sci.* 190 771–778. 10.1007/s11845-020-02328-x 32740716

[B4] Baird A. Samson S. (2014). Music evoked autobiographical memory after severe acquired brain injury: Preliminary findings from a case series. *Neuropsychol. Rehabil.* 24 125–143. 10.1080/09602011.2013.858642 24256344

[B5] Baker F. Tamplin J. Rickard N. Ponsford J. New P. Lee Y. C. (2019). A therapeutic songwriting intervention to promote reconstruction of self-concept and enhance well-being following brain or spinal cord injury: Pilot randomized controlled trial. *Clin. Rehabil.* 33 1045–1055. 10.1177/0269215519831417 30791702

[B6] Baker F. Wigram T. Gold C. (2005). The effects of a song-singing programme on the affective speaking intonation of people with traumatic brain injury. *Brain Inj.* 19 519–528. 10.1080/02699050400005150 16134740

[B7] Balduzzi S. Rücker G. Schwarzer G. (2019). How to perform a meta-analysis with R: A practical tutorial. *Evid. Based Ment. Health* 22 153–160. 10.1136/ebmental-2019-300117 31563865 PMC10231495

[B8] Barker T. Habibi N. Aromataris E. Stone J. Leonardi-Bee J. Sears K. (2024). The revised JBI critical appraisal tool for the assessment of risk of bias for quasi-experimental studies. *JBI Evid. Synth.* 22 378–388. 10.11124/JBIES-23-00268 38287725

[B9] Barker T. Stone J. Sears K. Klugar M. Tufanaru C. Leonardi-Bee J. (2023). The revised JBI critical appraisal tool for the assessment of risk of bias for randomized controlled trials. *JBI Evid. Synth.* 21 494–506. 10.11124/JBIES-22-00430 36727247

[B10] Baylan S. Haig C. MacDonald M. Stiles C. Easto J. Thomson M. (2020). Measuring the effects of listening for leisure on outcome after stroke (MELLO): A pilot randomized controlled trial of mindful music listening. *Int. J. Stroke* 15 149–158. 10.1177/1747493019841250 30940047 PMC7045280

[B11] Belfi A. Tranel D. (2014). Impaired naming of famous musical melodies is associated with left temporal polar damage. *Neuropsychology* 28 429–435. 10.1037/neu0000051 24364392 PMC4095894

[B12] Bernardi N. Cioffi M. Ronchi R. Maravita A. Bricolo E. Zigiotto L. (2017). Improving left spatial neglect through music scale playing. *J. Neuropsychol.* 11 135–158. 10.1111/jnp.12078 26146986

[B13] Bitan T. Simic T. Saverino C. Jones C. Glazer J. Collela B. (2018). Changes in resting-state connectivity following melody-based therapy in a patient with aphasia. *Neural Plast.* 2018:6214095. 10.1155/2018/6214095 29796017 PMC5896238

[B14] Bower J. Catroppa C. Grocke D. Shoemark H. (2014). Music therapy for early cognitive rehabilitation post-childhood TBI: An intrinsic mixed methods case study. *Dev. Neurorehabil.* 17 339–346. 10.3109/17518423.2013.778910 23815784

[B15] Bunketorp-Käll L. Lundgren-Nilsson Å Samuelsson H. Pekny T. Blomvé K. Pekna M. (2017). Long-term improvements after multimodal rehabilitation in late phase after *Stroke*: A randomized controlled trial. *Stroke* 48 1916–1924. 10.1161/STROKEAHA.116.016433 28619985

[B16] Carey L. M. (2012). *Stroke Rehabilitation: Insights from Neuroscience and Imaging.* Oxford: Oxford Academic.

[B17] Carrière M. Larroque S. Martial C. Bahri M. Aubinet C. Perrin F. (2020). An echo of consciousness: Brain function during preferred music. *Brain Connect.* 10 385–395. 10.1089/brain.2020.0744 32567335

[B18] Cavenaghi A. Mallah N. Navarro L. Martinón-Torres F. Gómez-Carballa A. Salas A. (2025). Decoding the peripheral transcriptomic and meta-genomic response to music in Autism Spectrum Disorder via saliva-based RNA sequencing. *BioRxiv* 10.1101/2025.1107.1104.663204 PMC12682756 41367385

[B19] Cha Y. Kim Y. Hwang S. Chung Y. (2014). Intensive gait training with rhythmic auditory stimulation in individuals with chronic hemiparetic stroke: A pilot randomized controlled study. *NeuroRehabilitation* 35 681–688. 10.3233/NRE-141182 25318784

[B20] Chong H. Cho S. Kim S. (2014). Hand rehabilitation using MIDI keyboard playing in adolescents with brain damage: A preliminary study. *NeuroRehabilitation* 34 147–155. 10.3233/NRE-131026 24270322

[B21] Colombo R. Raglio A. Panigazzi M. Mazzone A. Bazzini G. Imarisio C. (2019). The sonichand protocol for rehabilitation of hand motor function: A validation and feasibility study. *IEEE Trans. Neural Syst. Rehabil. Eng.* 27 664–672. 10.1109/TNSRE.2019.2905076 30872238

[B22] Conklyn D. Novak E. Boissy A. Bethoux F. Chemali K. (2012). The effects of modified melodic intonation therapy on nonfluent aphasia: A pilot study. *J. Speech Lang. Hear. Res.* 55 1463–1471. 10.1044/1092-4388(2012/11-0105) 22411278

[B23] Dayan E. Cohen L. (2011). Neuroplasticity subserving motor skill learning. *Neuron* 72 443–454. 10.1016/j.neuron.2011.10.008 22078504 PMC3217208

[B24] de Manzano Ö Ullén F. (2018). Same Genes, different brains: Neuroanatomical differences between monozygotic twins discordant for musical training. *Cereb. Cortex* 28 387–394. 10.1093/cercor/bhx299 29136105

[B25] DerSimonian R. Laird N. (1986). Meta-analysis in clinical trials. *Control Clin. Trials* 7 177–188. 10.1016/0197-2456(86)90046-2 3802833

[B26] Elsner B. Schöler A. Kon T. Mehrholz J. (2020). Walking with rhythmic auditory stimulation in chronic patients after stroke: A pilot randomized controlled trial. *Physiother. Res. Int*. 25:e1800. 10.1002/pri.1800 31237045

[B27] Fan L. Quijano-Ruiz A. Wang C. Zhao H. Wang D. Wu H. (2024). Effects of personalized music listening on post-stroke cognitive impairment: A randomized controlled trial. *Complement Ther. Clin. Pract.* 57:101885. 10.1016/j.ctcp.2024.101885 39098085

[B28] Ferreri L. Mas-Herrero E. Zatorre R. Ripollés P. Gomez-Andres A. Alicart H. (2019). Dopamine modulates the reward experiences elicited by music. *Proc. Natl. Acad. Sci. U S A.* 116 3793–3798. 10.1073/pnas.1811878116 30670642 PMC6397525

[B29] Fletcher J. Edberg A. Grifka R. Westendorp J. Elias A. Knott J. (2025). Music interventions in hyperacute and acute stroke patients: A randomized controlled pilot feasibility study. *Ann. Clin. Transl. Neurol.* 12 938–946. 10.1002/acn3.70024 40033585 PMC12093327

[B30] Friedman N. Chan V. Zondervan D. Bachman M. Reinkensmeyer D. (2011). MusicGlove: Motivating and quantifying hand movement rehabilitation by using functional grips to play music. *Annu. Int. Conf. IEEE Eng. Med. Biol. Soc.* 2011 2359–2363. 10.1109/IEMBS.2011.6090659 22254815

[B31] Fujioka T. Chen J. Black S. Chen J. Honjo K. Dawson D. (2025). Beta- and gamma-band neuromagnetic oscillations in chronic stroke rehabilitation using music-supported therapy and manual training. *Ann. N. Y. Acad. Sci.* 1552 362–372. 10.1111/nyas.70041 40930509

[B32] Fujioka T. Dawson D. Wright R. Honjo K. Chen J. Chen J. (2018). The effects of music-supported therapy on motor, cognitive, and psychosocial functions in chronic stroke. *Ann. N. Y. Acad. Sci.* 10.1111/nyas.13706 Online ahead of print. 29797585

[B33] GBD 2021 Nervous System Disorders Collaborators. (2024). Nervous system disorders collaborators, Global, regional, and national burden of disorders affecting the nervous system, 1990-2021: A systematic analysis for the Global burden of disease study 2021. *Lancet Neurol.* 23 344–381. 10.1016/S1474-4422(24)00038-3 38493795 PMC10949203

[B34] Ghai S. (2023). Does music therapy improve gait after traumatic brain injury and spinal cord injury? A mini systematic review and meta-analysis. *Brain Sci.* 13:522. 10.3390/brainsci13030522 36979332 PMC10046548

[B35] Giuliano C. Karahalios A. Neil C. Allen J. Levinger I. (2017). The effects of resistance training on muscle strength, quality of life and aerobic capacity in patients with chronic heart failure - A meta-analysis. *Int. J. Cardiol.* 227 413–423. 10.1016/j.ijcard.2016.11.023 27843045

[B36] Gómez-Carballa A. Navarro L. Pardo-Seco J. Bello X. Pischedda S. Viz-Lasheras S. (2023). Music compensates for altered gene expression in age-related cognitive disorders. *Sci. Rep*. 13:21259. 10.1038/s41598-023-48094-5 38040763 PMC10692168

[B37] Grau-Sánchez J. Amengual J. Rojo N. Veciana de Las Heras M. Montero J. Rubio F. (2013). Plasticity in the sensorimotor cortex induced by Music-supported therapy in stroke patients: A TMS study. *Front. Hum. Neurosci.* 7:494. 10.3389/fnhum.2013.00494 24027507 PMC3759754

[B38] Grau-Sánchez J. Duarte E. Ramos-Escobar N. Sierpowska J. Rueda N. Redón S. (2018). Music-supported therapy in the rehabilitation of subacute stroke patients: A randomized controlled trial. *Ann. N. Y. Acad. Sci.* 10.1111/nyas.13590 Online ahead of print. 29607506

[B39] Grau-Sánchez J. Münte T. Altenmüller E. Duarte E. Rodríguez-Fornells A. (2020). Potential benefits of music playing in stroke upper limb motor rehabilitation. *Neurosci. Biobehav. Rev.* 112 585–599. 10.1016/j.neubiorev.2020.02.027 32092314

[B40] Grau-Sánchez J. Ramos N. Duarte E. Särkämö T. Rodríguez-Fornells A. (2017). Time course of motor gains induced by music-supported therapy after stroke: An exploratory case study. *Neuropsychology* 31 624–635. 10.1037/neu0000355 28406666

[B41] Hayden R. Clair A. Johnson G. Otto D. (2009). The effect of rhythmic auditory stimulation (RAS) on physical therapy outcomes for patients in gait training following stroke: A feasibility study. *Int. J. Neurosci.* 119 2183–2195. 10.3109/00207450903152609 19916847

[B42] Higgins J. P. Green S. (2011). *Cochrane Handbook for Systematic Reviews of Interventions Version 5.1.0.* London: The Cochrane Collaboration.

[B43] Hosp J. Pekanovic A. Rioult-Pedotti M. Luft A. (2011). Dopaminergic projections from midbrain to primary motor cortex mediate motor skill learning. *J. Neurosci.* 31 2481–2487. 10.1523/JNEUROSCI.5411-10.2011 21325515 PMC6623715

[B44] Hutchinson K. Sloutsky R. Collimore A. Adams B. Harris B. Ellis T. (2020). A music-based digital therapeutic: Proof-of-concept automation of a progressive and Individualized rhythm-based walking training program after stroke. *Neurorehabil. Neural Repair.* 34 986–996. 10.1177/1545968320961114 33040685

[B45] Jeong E. Ham Y. Lee S. Shin J. (2024). Virtual reality-based music attention training for acquired brain injury: A randomized crossover study. *Ann. N. Y. Acad. Sci.* 1541 151–162. 10.1111/nyas.15249 39476208 PMC11580773

[B46] Jeong E. Ryu H. Shin J. Kwon G. Jo G. Lee J. (2018). High oxygen exchange to music indicates auditory distractibility in acquired brain injury: An fNIRS study with a vector-based phase analysis. *Sci. Rep.* 8:16737. 10.1038/s41598-018-35172-2 30425287 PMC6233191

[B47] Jeong S. Kim M. (2007). Effects of a theory-driven music and movement program for stroke survivors in a community setting. *Appl. Nurs. Res.* 20 125–131. 10.1016/j.apnr.2007.04.005 17693215

[B48] Jerde T. Childs S. Handy S. Nagode J. Pardo J. (2011). Dissociable systems of working memory for rhythm and melody. *Neuroimage* 57 1572–1579. 10.1016/j.neuroimage.2011.05.061 21645625

[B49] Jones C. Richard N. Thaut M. (2021). Investigating music-based cognitive rehabilitation for individuals with moderate to severe chronic acquired brain injury: A feasibility experiment. *NeuroRehabilitation* 48 209–220. 10.3233/NRE-208015 33664158

[B50] Jun E. Roh Y. Kim M. (2013). The effect of music-movement therapy on physical and psychological states of stroke patients. *J. Clin. Nurs.* 22 22–31. 10.1111/j.1365-2702.2012.04243.x 22978325

[B51] Jungblut M. Mais C. Binkofski F. Schüppen A. (2022). The efficacy of a directed rhythmic-melodic voice training in the treatment of chronic non-fluent aphasia-behavioral and imaging results. *J. Neurol.* 269 5070–5084. 10.1007/s00415-022-11163-2 35604466

[B52] Kasdan A. Kiran S. (2018). Please don’t stop the music: Song completion in patients with aphasia. *J. Commun. Disord*. 75, 72–86. 10.1016/j.jcomdis.2018.06.005 30031236

[B53] Kim D. Park Y. Choi J. Im S. Jung K. Cha Y. (2011). Effects of music therapy on mood in stroke patients. *Yonsei Med. J.* 52 977–981. 10.3349/ymj.2011.52.6.977 22028163 PMC3220261

[B54] Kim S. Shin Y. Jeong E. Cho S. (2023). Movement-specific keyboard playing for hand function in adolescents and young adults with acquired brain injury. *Front. Neurol.* 13:1062615. 10.3389/fneur.2022.1062615 36698898 PMC9868739

[B55] Koelsch S. (2015). Music-evoked emotions: Principles, brain correlates, and implications for therapy. *Ann. N. Y. Acad. Sci.* 1337 193–201. 10.1111/nyas.12684 25773635

[B56] Lin S. Yang P. Lai C. Su Y. Yeh Y. Huang M. (2011). Mental health implications of music: Insight from neuroscientific and clinical studies. *Harv. Rev. Psychiatry* 19 34–46. 10.3109/10673229.2011.549769 21250895

[B57] Liu X. Kanduri C. Oikkonen J. Karma K. Raijas P. Ukkola-Vuoti L. (2016). Detecting signatures of positive selection associated with musical aptitude in the human genome. *Sci. Rep.* 6:21198. 10.1038/srep21198 26879527 PMC4754774

[B58] Loetscher T. Potter K. Wong D. das Nair R. (2019). Cognitive rehabilitation for attention deficits following stroke. *Cochrane Database Syst. Rev.* 2019:CD002842. 10.1002/14651858.CD002842.pub3 31706263 PMC6953353

[B59] Lynch C. LaGasse A. (2016). Training endogenous task shifting using music therapy: A feasibility study. *J. Music Ther.* 53 279–307. 10.1093/jmt/thw008 27235114

[B60] Maas A. I. R. Menon D. K. Manley G. T. Abrams M. Akerlund C. Andelic N. (2022). Traumatic brain injury: Progress and challenges in prevention, clinical care, and research. *Lancet Neurol.* 21 1004–1060. 10.1016/S1474-4422(22)00309-X 36183712 PMC10427240

[B61] Magee W. Clark I. Tamplin J. Bradt J. (2017). Music interventions for acquired brain injury. *Cochrane Database Syst. Rev.* 1:CD006787. 10.1002/14651858.CD006787.pub3 28103638 PMC6464962

[B62] Marchina S. Norton A. Schlaug G. (2023). Effects of melodic intonation therapy in patients with chronic nonfluent aphasia. *Ann. N. Y. Acad. Sci.* 1519 173–185. 10.1111/nyas.14927 36349876 PMC10262915

[B63] Martínez-Molina N. Siponkoski S. Kuusela L. Laitinen S. Holma M. Ahlfors M. (2021). Resting-state network plasticity induced by music therapy after traumatic brain injury. *Neural Plast.* 2021:6682471. 10.1155/2021/6682471 33763126 PMC7964116

[B64] Mazhari-Jensen D. S. Jacobsen S. L. Jespersen K. V. (2023). Inpatient stroke survivors with low gait functioning benefit from music interventions during cardiorespiratory exercise: A randomized cross-over trial. *NJMT* 32 462–481. 10.1080/08098131.2023.2190403

[B65] Mehr S. Singh M. Knox D. Ketter D. Pickens-Jones D. Atwood S. (2019). Universality and diversity in human song. *Science* 366:eaax0868. 10.1126/science.aax0868 31753969 PMC7001657

[B66] Mishra R. Florez-Perdomo W. Shrivatava A. Chouksey P. Raj S. Moscote-Salazar L. (2021). Role of music therapy in traumatic brain injury: A systematic review and meta-analysis. *World Neurosurg.* 146 197–204. 10.1016/j.wneu.2020.10.130 33130286

[B67] Monroe P. Halaki M. Kumfor F. Ballard K. (2020). The effects of choral singing on communication impairments in acquired brain injury: A systematic review. *Int. J. Lang. Commun. Disord.* 55 303–319. 10.1111/1460-6984.12527 32096327

[B68] Murakami T. Hama S. Yamashita H. Onoda K. Hibino S. Sato H. (2014). Neuroanatomic pathway associated with attentional deficits after stroke. *Brain Res.* 1544 25–32. 10.1016/j.brainres.2013.11.029 24321618

[B69] Navarro L. Gómez-Carballa A. Pischedda S. Montoto-Louzao J. Viz-Lasheras S. Camino-Mera A. (2023). Sensogenomics of music and Alzheimer’s disease: An interdisciplinary view from neuroscience, transcriptomics, and epigenomics. *Front. Aging Neurosci.* 15:1063536. 10.3389/fnagi.2023.1063536 36819725 PMC9935844

[B70] Navarro L. Mallah N. Nowak W. Pardo-Seco J. Gómez-Carballa A. Pischedda S. (2025). The effect of music interventions in autism spectrum disorder: A systematic review and meta-analysis. *Front. Integr. Neurosci.* 12:10.3389/fnint.2025.1673618. 10.1101/2025.07.03.25330837 PMC12602440 41230119

[B71] Navarro L. Martinón-Torres F. Salas A. (2021). Sensogenomics and the biological background underlying musical stimuli: Perspectives for a new era of musical research. *Genes (Basel)* 12:1454. 10.3390/genes12091454 34573436 PMC8472585

[B72] Nayak S. Wheeler B. L. Shiflett S. C. Agostinelli S. (2000). Effect of music therapy on mood and social interaction among individuals with acute traumatic brain injury and stroke. *Rehabil. Psychol.* 45 274–283. 10.1037/0090-5550.45.3.274

[B73] Nikmaram N. Scholz D. Großbach M. Schmidt S. Spogis J. Belardinelli P. (2019). Musical sonification of arm movements in stroke rehabilitation yields limited benefits. *Front. Neurosci.* 13:1378. 10.3389/fnins.2019.01378 31920526 PMC6933006

[B74] Norton A. Zipse L. Marchina S. Schlaug G. (2009). Melodic intonation therapy: Shared insights on how it is done and why it might help. *Ann. N. Y. Acad. Sci.* 1169 431–436. 10.1111/j.1749-6632.2009.04859.x 19673819 PMC2780359

[B75] Okumura Y. Asano Y. Takenaka S. Fukuyama S. Yonezawa S. Kasuya Y. (2014). Brain activation by music in patients in a vegetative or minimally conscious state following diffuse brain injury. *Brain Inj* 28 944–950. 10.3109/02699052.2014.888477 24655034

[B76] Omigie D. Samson S. A. (2014). protective effect of musical expertise on cognitive outcome following brain damage? *Neuropsychol. Rev.* 24 445–460. 10.1007/s11065-014-9274-5 25380766

[B77] Page M. J. McKenzie J. E. Bossuyt P. M. Boutron I. Hoffmann T. C. Mulrow C. D. (2021). The PRISMA 2020 statement: An updated guideline for reporting systematic reviews. *Rev. Esp. Cardiol.* 74 790–799. 34446261 10.1016/j.rec.2021.07.010

[B78] Palmquist E. T. Underbjerg M. Ridder H. M. Jespersen K. V. (2025). Music listening for improvement of sleep in post-acute rehabilitation of adults with acquired brain injury: A feasibility study. *NJMT* 34 245–260. 10.1080/08098131.2024.2396111

[B79] Palumbo A. Aluru V. Battaglia J. Geller D. Turry A. Ross M. (2022). Music upper limb therapy-integrated provides a feasible enriched environment and reduces post-stroke depression: A pilot randomized controlled trial. *Am. J. Phys. Med. Rehabil.* 101 937–946. 10.1097/PHM.0000000000001938 34864768 PMC9163211

[B80] Pantev C. Herholz S. (2011). Plasticity of the human auditory cortex related to musical training. *Neurosci Biobehav Rev.* 35 2140–2154. 10.1016/j.neubiorev.2011.06.010 21763342

[B81] Park I. M. Oh D. W. Kim S. Y. Choi J. D. (2010). Clinical feasibility of integrating fast-tempoauditory stimulation with self-adopted walking training for improving walking function in post-stroke patients: A randomized, controlled pilot trial. *J. Phys. Ther. Sci.* 22 295–300. 10.1589/jpts.22.295

[B82] Park S. Williams R. Lee D. (2016). Effect of preferred music on agitation after traumatic brain injury. *West J. Nurs. Res.* 38 394–410. 10.1177/0193945915593180 26129873

[B83] Patel A. (2011). Why would musical training benefit the neural encoding of speech? The OPERA hypothesis. *Front. Psychol.* 2:142. 10.3389/fpsyg.2011.00142 21747773 PMC3128244

[B84] Pearson M. Smart N. (2018). Reported methods for handling missing change standard deviations in meta-analyses of exercise therapy interventions in patients with heart failure: A systematic review. *PLoS One* 13:e0205952. 10.1371/journal.pone.0205952 30335861 PMC6193694

[B85] Peyre I. Roby-Brami A. Segalen M. Giron A. Caramiaux B. Marchand-Pauvert V. (2023). Effect of sonification types in upper-limb movement: A quantitative and qualitative study in hemiparetic and healthy participants. *J. Neuroeng. Rehabil.* 20:136. 10.1186/s12984-023-01248-y 37798637 PMC10552218

[B86] Posner M. Rothbart M. Sheese B. Voelker P. (2014). Developing attention: Behavioral and brain mechanisms. *Adv. Neurosci.* 2014:405094. 10.1155/2014/405094 25110757 PMC4125572

[B87] Raghavan P. Geller D. Guerrero N. Aluru V. Eimicke J. P. Teresi J. A. (2016). Music upper limb therapy-integrated: An enriched collaborative approach for stroke rehabilitation. *Front. Hum. Neurosci*. 10:498. 10.3389/fnhum.2016.00498 27774059 PMC5053999

[B88] Raglio A. Oasi O. Gianotti M. Rossi A. Goulene K. Stramba-Badiale M. (2016). Improvement of spontaneous language in stroke patients with chronic aphasia treated with music therapy: A randomized controlled trial. *Int. J. Neurosci.* 126 235–242. 10.3109/00207454.2015.1010647 26000622

[B89] Raglio A. Panigazzi M. Colombo R. Tramontano M. Iosa M. Mastrogiacomo S. (2021). Hand rehabilitation with sonification techniques in the subacute stage of stroke. *Sci. Rep.* 11:7237. 10.1038/s41598-021-86627-y 33790343 PMC8012636

[B90] Raglio A. Zaliani A. Baiardi P. Bossi D. Sguazzin C. Capodaglio E. (2017). Active music therapy approach for stroke patients in the post-acute rehabilitation. *Neurol. Sci.* 38 893–897. 10.1007/s10072-017-2827-7 28138867

[B91] Ribeiro A. Ramos A. Bermejo E. Casero M. Corrales J. Grantham S. (2014). Effects of different musical stimuli in vital signs and facial expressions in patients with cerebral damage: A pilot study. *J. Neurosci. Nurs.* 46 117–124. 10.1097/JNN.0000000000000037 24556659

[B92] Ripollés P. Rojo N. Grau-Sánchez J. Amengual J. Càmara E. Marco-Pallarés J. (2016). Music supported therapy promotes motor plasticity in individuals with chronic stroke. *Brain Imaging Behav.* 10 1289–1307. 10.1007/s11682-015-9498-x 26707190

[B93] Rojo N. Amengual J. Juncadella M. Rubio F. Camara E. Marco-Pallares J. (2011). Music-supported therapy induces plasticity in the sensorimotor cortex in chronic stroke: A single-case study using multimodal imaging (fMRI-TMS). *Brain Inj* 25 787–793. 10.3109/02699052.2011.576305 21561296

[B94] Salas A. Navarro L. Martinón-Torres F. Gómez-Carballa A. (2025). Beyond behavioral studies: Exploring the multi-omics impact of music on human health. *Phys. Life Rev*. 10.1016/j.plrev.2025.1009.1002 41365103

[B95] Salimpoor V. Benovoy M. Larcher K. Dagher A. Zatorre R. (2011). Anatomically distinct dopamine release during anticipation and experience of peak emotion to music. *Nat. Neurosci.* 14 257–262. 10.1038/nn.2726 21217764

[B96] Salimpoor V. Benovoy M. Longo G. Cooperstock J. Zatorre R. (2009). The rewarding aspects of music listening are related to degree of emotional arousal. *PLoS One* 4:e7487. 10.1371/journal.pone.0007487 19834599 PMC2759002

[B97] Salimpoor V. Zald D. Zatorre R. Dagher A. McIntosh A. (2015). Predictions and the brain: How musical sounds become rewarding. *Trends Cogn Sci.* 19 86–91. 10.1016/j.tics.2014.12.001 25534332

[B98] Särkämö T. Soto D. (2012). Music listening after stroke: Beneficial effects and potential neural mechanisms. *Ann. N. Y. Acad. Sci.* 1252 266–281. 10.1111/j.1749-6632.2011.06405.x 22524369

[B99] Särkämö T. Pihko E. Laitinen S. Forsblom A. Soinila S. Mikkonen M. (2010). Music and speech listening enhance the recovery of early sensory processing after stroke. *J. Cogn. Neurosci.* 22 2716–2727. 10.1162/jocn.2009.21376 19925203

[B100] Särkämö T. Ripollés P. Vepsäläinen H. Autti T. Silvennoinen H. Salli E. (2014). Structural changes induced by daily music listening in the recovering brain after middle cerebral artery stroke: A voxel-based morphometry study. *Front. Hum. Neurosci.* 8:245. 10.3389/fnhum.2014.00245 24860466 PMC4029020

[B101] Särkämö T. Tervaniemi M. Laitinen S. Forsblom A. Soinila S. Mikkonen M. (2008). Music listening enhances cognitive recovery and mood after middle cerebral artery stroke. *Brain* 131 866–876. 10.1093/brain/awn013 18287122

[B102] Schauer M. Mauritz K. (2003). Musical motor feedback (MMF) in walking hemiparetic stroke patients: Randomized trials of gait improvement. *Clin. Rehabil.* 17 713–722. 10.1191/0269215503cr668oa 14606736

[B103] Schneider S. Münte T. F. Rodríguez-Fornells A. Sailer M. Altenmüller E. (2010). Music-supported training is more efficient than functional motor training for recovery of fine motor skills in stroke patients. *Music Percept.* 27 271–280. 10.1525/mp.2010.27.4.271

[B104] Schneider S. Schönle P. Altenmüller E. Münte T. (2007). Using musical instruments to improve motor skill recovery following a stroke. *J. Neurol.* 254 1339–1346. 10.1007/s00415-006-0523-2 17260171

[B105] Scholz D. Rhode S. Großbach M. Rollnik J. Altenmüller E. (2015). Moving with music for stroke rehabilitation: A sonification feasibility study. *Ann. N. Y. Acad. Sci.* 1337 69–76. 10.1111/nyas.12691 25773619

[B106] Scholz D. Rohde S. Nikmaram N. Brückner H. Großbach M. Rollnik J. (2016). Sonification of arm movements in stroke rehabilitation - A novel approach in neurologic music therapy. *Front. Neurol.* 7:106. 10.3389/fneur.2016.00106 27445970 PMC4928599

[B107] Schulze K. Zysset S. Mueller K. Friederici A. Koelsch S. (2011). Neuroarchitecture of verbal and tonal working memory in nonmusicians and musicians. *Hum, Brain Mapp.* 32 771–783. 10.1002/hbm.21060 20533560 PMC6870416

[B108] Segura E. Grau-Sánchez J. Cerda-Company X. Porto M. De la Cruz-Puebla M. Sanchez-Pinsach D. (2024). Enriched music-supported therapy for individuals with chronic stroke: A randomized controlled trial. *J. Neurol.* 271 6606–6617. 10.1007/s00415-024-12570-3 39112892

[B109] Segura E. Grau-Sánchez J. Sanchez-Pinsach D. De la Cruz M. Duarte E. Arcos J. L. (2021). Designing an app for home-based enriched Music-supported Therapy in the rehabilitation of patients with chronic stroke: A pilot feasibility study. *Brain Inj.* 35 1585–1597. 10.1080/02699052.2021.1975819 34554859

[B110] Seibert P. Fee L. Basom J. Zimmerman C. (2000). Music and the brain: The impact of music on an oboist’s fight for recovery. *Brain Inj* 14 295–302. 10.1080/026990500120763 10759046

[B111] Shea B. Reeves B. Wells G. Thuku M. Hamel C. Moran J. (2017). AMSTAR 2: A critical appraisal tool for systematic reviews that include randomised or non-randomised studies of healthcare interventions, or both. *BMJ* 358:j4008. 10.1136/bmj.j4008 28935701 PMC5833365

[B112] Sheridan C. Thaut C. Brooks D. Patterson K. (2021). Feasibility of a rhythmic auditory stimulation gait training program in community-dwelling adults after TBI: A case report. *NeuroRehabilitation* 48 221–230. 10.3233/NRE-208016 33664159

[B113] Sihvonen A. Leo V. Ripollés P. Lehtovaara T. Ylönen A. Rajanaro P. (2020). Vocal music enhances memory and language recovery after stroke: Pooled results from two RCTs. *Ann. Clin. Transl. Neurol.* 7 2272–2287. 10.1002/acn3.51217 33022148 PMC7664275

[B114] Sihvonen A. Sammler D. Ripollés P. Leo V. Rodríguez-Fornells A. Soinila S. (2022a). Right ventral stream damage underlies both poststroke aprosodia and amusia. *Eur. J. Neurol.* 29 873–882. 10.1111/ene.15148 34661326

[B115] Sihvonen A. Särkämö T. Leo V. Tervaniemi M. Altenmüller E. Soinila S. (2017a). Music-based interventions in neurological rehabilitation. *Lancet Neurol.* 16 648–660. 10.1016/S1474-4422(17)30168-0 28663005

[B116] Sihvonen A. Särkämö T. Ripollés P. Leo V. Saunavaara J. Parkkola R. (2017b). Functional neural changes associated with acquired amusia across different stages of recovery after stroke. *Sci. Rep.* 7:11390. 10.1038/s41598-017-11841-6 28900231 PMC5595783

[B117] Sihvonen A. Siponkoski S. Martínez-Molina N. Laitinen S. Holma M. Ahlfors M. (2022b). Neurological music therapy rebuilds structural connectome after traumatic brain injury: Secondary analysis from a randomized controlled trial. *J. Clin. Med.* 11:2184. 10.3390/jcm11082184 35456277 PMC9032739

[B118] Siponkoski S. Koskinen S. Laitinen S. Holma M. Ahlfors M. Jordan-Kilkki P. (2022). Effects of neurological music therapy on behavioural and emotional recovery after traumatic brain injury: A randomized controlled cross-over trial. *Neuropsychol. Rehabil.* 32 1356–1388. 10.1080/09602011.2021.1890138 33657970

[B119] Siponkoski S. Martínez-Molina N. Kuusela L. Laitinen S. Holma M. Ahlfors M. (2020). Music therapy enhances executive functions and prefrontal structural neuroplasticity after traumatic brain injury: Evidence from a randomized controlled trial. *J. Neurotrauma* 37 618–634. 10.1089/neu.2019.6413 31642408

[B120] Siponkoski S. Pitkäniemi A. Laitinen S. Särkämö E. Pentikäinen E. Eloranta H. (2023). Efficacy of a multicomponent singing intervention on communication and psychosocial functioning in chronic aphasia: A randomized controlled crossover trial. *Brain Commun.* 5:fcac337. 10.1093/braincomms/fcac337 36687394 PMC9847537

[B121] Street A. Magee W. Bateman A. Parker M. Odell-Miller H. Fachner J. (2018). Home-based neurologic music therapy for arm hemiparesis following stroke: Results from a pilot, feasibility randomized controlled trial. *Clin. Rehabil.* 32 18–28. 10.1177/0269215517717060 28643570 PMC5751852

[B122] Suh J. H. Han S. J. Jeon S. Y. Kim H. J. Lee J. E. Yoon T. S. (2014). Effect of rhythmic auditory stimulation on gait and balance in hemiplegic stroke patients. *NeuroRehabilitation* 34, 193–199. 10.3233/NRE-131008 24284453

[B123] Talsma D. Senkowski D. Soto-Faraco S. Woldorff M. (2010). The multifaceted interplay between attention and multisensory integration. *Trends Cogn. Sci.* 14 400–410. 10.1016/j.tics.2010.06.008 20675182 PMC3306770

[B124] Tamplin J. Baker F. Jones B. Way A. Lee S. (2013). ‘Stroke a Chord’: The effect of singing in a community choir on mood and social engagement for people living with aphasia following a stroke. *NeuroRehabilitation* 32 929–941. 10.3233/NRE-130916 23867418

[B125] Thaut M. Gardiner J. Holmberg D. Horwitz J. Kent L. Andrews G. (2009). Neurologic music therapy improves executive function and emotional adjustment in traumatic brain injury rehabilitation. *Ann. N. Y. Acad. Sci.* 1169 406–416. 10.1111/j.1749-6632.2009.04585.x 19673815

[B126] Thaut M. Leins A. Rice R. Argstatter H. Kenyon G. McIntosh G. (2007). Rhythmic auditory stimulation improves gait more than NDT/Bobath training in near-ambulatory patients early poststroke: A single-blind, randomized trial. *Neurorehabil. Neural Repair.* 21 455–459. 10.1177/1545968307300523 17426347

[B127] Thompson S. Hays K. Weintraub A. Ketchum J. Kowalski R. (2021). Rhythmic auditory stimulation and gait training in traumatic brain injury: A pilot study. *J. Music Ther.* 58 70–94. 10.1093/jmt/thaa016 33095230

[B128] Tong Y. Forreider B. Sun X. Geng X. Zhang W. Du H. (2015). Music-supported therapy (MST) in improving post-stroke patients’ upper-limb motor function: A randomised controlled pilot study. *Neurol. Res.* 37 434–440. 10.1179/1743132815Y.0000000034 25916420

[B129] Ueda M. Hayashi K. Suzuki A. Nakaya Y. Takaku N. Miura T. (2024). Treatment of subcortical aphasia due to putaminal hemorrhage with the japanese version of melodic intonation therapy (MIT-J). *Cureus* 16:e55590. 10.7759/cureus.55590 38576684 PMC10994653

[B130] Ushasree B. Al Anzari A. Sampath Kumar N. Phanisree P. Indira S. Moscote-Salazar L. (2021). Post music session real-time EEG changes in patients who underwent neurosurgical intervention for neuronal dysfunction. *Neurol India* 69 1024–1026. 10.4103/0028-3886.323889 34507435

[B131] van der Meulen I. van de Sandt-Koenderman W. M. Heijenbrok-Kal M. H. Visch-Brink E. G. Ribbers G. M. (2014). The efficacy and timing of melodic intonation therapy in subacute aphasia. *Neurorehabil. Neural. Repair.* 28 536–544. 10.1177/1545968313517753 24449708

[B132] Van Vugt F. Ritter J. Rollnik J. Altenmüller E. (2014). Music-supported motor training after stroke reveals no superiority of synchronization in group therapy. *Front. Hum. Neurosci.* 8:315. 10.3389/fnhum.2014.00315 24904358 PMC4033001

[B133] Vaudreuil R. Nordstrom M. DeGraba T. Pasquina P. (2024). The role of technology in music therapy, occupational therapy, and co-treatment of an injured United States service member. *NJMT* 34 13–30. 10.1080/08098131.2024.2397795

[B134] Vik B. Skeie G. Vikane E. Specht K. (2018). Effects of music production on cortical plasticity within cognitive rehabilitation of patients with mild traumatic brain injury. *Brain Inj.* 32 634–643. 10.1080/02699052.2018.1431842 29388854

[B135] Villeneuve M. Lamontagne A. (2013). Playing piano can improve upper extremity function after stroke: Case studies. *Stroke Res. Treat.* 2013:159105. 10.1155/2013/159105 23533954 PMC3596897

[B136] Villeneuve M. Penhune V. Lamontagne A. (2014). A piano training program to improve manual dexterity and upper extremity function in chronic stroke survivors. *Front. Hum. Neurosci.* 8:662. 10.3389/fnhum.2014.00662 25202258 PMC4141215

[B137] Wan C. Rüber T. Hohmann A. Schlaug G. (2010). The therapeutic effects of singing in neurological disorders. *Music Percept.* 27 287–295. 10.1525/mp.2010.27.4.287 21152359 PMC2996848

[B138] Wang Y. Pan W. Li F. Ge J. Zhang X. Luo X. (2021). Effect of rhythm of music therapy on gait in patients with stroke. *J. Stroke Cerebrovasc. Dis.* 30:105544. 10.1016/j.jstrokecerebrovasdis.2020.105544 33341022

[B139] Welch G. Biasutti M. MacRitchie J. McPherson G. Himonides E. (2020). Editorial: The impact of music on human development and well-being. *Front. Psychol.* 11:1246. 10.3389/fpsyg.2020.01246 32625147 PMC7315798

[B140] Yang Y. Fang Y. Gao J. Geng G. (2019). Effects of five-element music on language recovery in patients with poststroke aphasia: A systematic review and meta-analysis. *J. Altern. Complement Med.* 25 993–1004. 10.1089/acm.2018.0479 31298550

[B141] Zatorre R. Salimpoor V. (2013). From perception to pleasure: Music and its neural substrates. *Proc. Natl. Acad. Sci. U S A.* 110 10430–10437. 10.1073/pnas.1301228110 23754373 PMC3690607

[B142] Zondervan D. Friedman N. Chang E. Zhao X. Augsburger R. Reinkensmeyer D. (2016). Home-based hand rehabilitation after chronic stroke: Randomized, controlled single-blind trial comparing the MusicGlove with a conventional exercise program. *J. Rehabil. Res. Dev.* 53 457–472. 10.1682/JRRD.2015.04.0057 27532880

[B143] Zumbansen A. Peretz I. Anglade C. Bilodeau J. Généreux S. Hubert M. (2017). Effect of choir activity in the rehabilitation of aphasia: A blind, randomised, controlled pilot study. *Aphasiology* 31 879–900. 10.1080/02687038.2016.1227424

